# Pyrolysis Bio-Oil as a Partial Substitute for Conventional Bitumen in More Sustainable Asphalt Mixtures

**DOI:** 10.3390/ma19214556

**Published:** 2026-10-28

**Authors:** Blanca Nélida Bautista-Layme, Adriana H. Martinez, Teresa López-Montero

**Affiliations:** Department of Civil and Environmental Engineering, Universitat Politècnica de Catalunya, 08034 Barcelona, Spain; blanca.nelida.bautista@upc.edu (B.N.B.-L.); adriana.martinez@upc.edu (A.H.M.)

**Keywords:** bio-binder, bituminous mixtures, RTFOT ageing, indirect tensile strength (*ITS*), technical feasibility

## Abstract

The use of bio-binders has sparked increasing interest in the production of bituminous mixtures for road pavements. For this reason, this article evaluates the effect of partially replacing two conventional 35/50 and 50/70 penetration-grade bitumens with bio-oil obtained from the pyrolysis of biomass. In the first stage, the bio-oil was subjected to thermal pre-treatment combined with agitation and conditioned at different temperatures and stirring speeds for different durations to progressively reduce the mass loss measured under thermogravimetric test conditions and achieve a homogeneous mixture with the base binder. Based on this pre-treatment, thirteen bio-binder formulations were initially evaluated using 35/50 bitumen and varying the bio-oil content to assess the properties of the bio-binders before and after short-term ageing. Subsequently, a gap-graded bituminous mixture, type BBTM 11B, was produced using the bio-binders, and the volumetric and mechanical properties were analysed to identify the bio-binder exhibiting the most suitable performance. Finally, the selected 10% bio-oil replacement level was evaluated using 50/70 bitumen. The results confirmed that the selected formulation satisfied the specific laboratory acceptance criteria evaluated for BBTM 11B mixtures.

## 1. Introduction and Objectives

Road pavement construction offers opportunities to improve sustainability through the recovery and reuse of by-products and waste from industrial, agricultural or urban sources. Among these waste streams, agricultural waste is of particular importance, as it accounts for approximately 60% of global biomass resources [1,2]. The valorisation of these materials in transport infrastructure may reduce reliance on virgin raw materials, promote resource efficiency, and contribute to more sustainable construction practices.

It is estimated that around 95% of the world’s roads are constructed using flexible pavements [3,4]. Flexible pavements commonly contain asphalt mixtures bound with bitumen, a product of petroleum refining derived from a non-renewable resource with a substantial carbon footprint [5,6]. It is therefore imperative to seek alternative and renewable sources of binders that will reduce dependence on oil [5,7].

The development of alternative binders has emerged as a key strategy for reducing dependence on conventional petroleum-based bitumen [8]. Two main approaches have been considered to reduce the use of traditional asphalt binders. The first involves the physical or chemical modification of bitumen through the incorporation of materials such as nanoclays, polymers, coffee grounds, or rubber powder, amongst others. The second approach utilises bio-binders, some of which are derived from pyrolysis or from resins, which have demonstrated performance comparable to that of conventional binders [4]; current research is focusing on addition rates of less than 15% [9].

The raw materials available for the production of bio-oils include biomass of agricultural, forestry, industrial and urban origin [10,11]. In the technical literature on pavements, the chemical composition of these bio-oils is compared with the hydrocarbon matrix of conventional bituminous binders, which is characterised by a high concentration of carbon (80–88%), hydrogen (8–11%), and oxygen (0.9–1.72%) [8,12,13,14,15,16,17,18]. In contrast, bio-oils exhibit a markedly different elemental profile, characterised by a lower carbon content and a higher oxygen content [4,5,11,12,19,20,21,22,23].

The process of incorporating bio-oil into the bituminous binder varies substantially depending on the origin of the biomass and its function as a modifier, rejuvenator or extender [1,5,6,7,24,25]. For woody biomass (such as waste wood, pine, cedar and sawdust), fast pyrolysis is the most commonly reported production technique [7,12,26,27,28,29]. Bio-oils derived from this group are typically used at substitution levels of 2 wt% to 30 wt%, requiring temperatures for mixing with bitumen ranging from 120 °C to 150 °C, high shear rates of 1000 rpm to 5000 rpm and mixing times of 15 min to 60 min [9,12,27,28,29,30,31,32,33,34,35,36].

In the case of organic and industrial waste (including used vegetable oils, maize stubble, sugarcane bagasse, swine manure, and waste engine oils), the incorporation rates are often between 1 wt% and 20 wt% [4,9,37,38,39,40,41,42,43,44]. A key factor identified in the literature for the use of these bio-oils is their free moisture content, which typically ranges from 15 wt% to 30 wt% depending on the source and the refining process [13,18,20,37,45,46,47,48,49,50,51,52]. As the thermal mixing process takes place at temperatures between 120 °C and 180 °C, it is essential to control the water content in the bio-oil. This control prevents sudden thermal changes during mixing and ensures adequate workability, promoting the homogeneous incorporation of the modifier into the conventional binder [9,14,18,20,26,36,38,39,40,41,42,43,44,49,53].

Pyrolysis involves the thermal degradation of biomass in the absence of oxygen. Pyrolysis temperatures depend on the process and feedstock; temperatures of around 500–550 °C are reported for some systems [54,55,56]. This process simultaneously generates a three-phase system comprising a solid fraction (biochar), a liquid fraction (bio-oil) and a gaseous fraction (non-condensable gases or NCG) [13,30]. The commercial scale of waste biomass pyrolysis is expanding rapidly, with global production projected to reach 9.3 million tonnes by 2030. The maturation of this sector is driving the comprehensive valorisation of its co-products, sparking significant interest in the use of bio-oil as a rejuvenating additive in the asphalt industry. Consequently, its transition into road infrastructure requires rigorous physical characterisation to ensure the technical viability of these bio-binders [31].

The partial replacement of petroleum-derived bitumen with biomass-derived materials represents a promising strategy for improving resource efficiency and promoting circular-economy principles in road pavement construction. In particular, bio-oil obtained from the pyrolysis of residual agricultural biomass offers the possibility of valorising biomass while reducing the consumption of conventional petroleum-derived bitumen. Previous studies have highlighted the potential environmental benefits associated with bio-oil incorporation in asphalt mixtures [57,58].

Bio-oils are organic fluids with a high content of oxygenated compounds that remain in a liquid state under ambient conditions [2,59,60,61]. In their crude state, they typically contain between 10% and 30% by weight of water, and may even reach higher values in some cases [62], which can adversely affect their stability and performance when used as binders [4,63]. Furthermore, they contain a wide variety of oxygenated organic compounds, some of which are highly reactive and can trigger chemical instability during storage, increasing the water content and altering their properties.

In terms of mechanical benefits, some studies have reported improvements in fatigue behaviour or workability under specific conditions [19,45], although the response depends on the material and dosage. The main challenge with bio-binders concerns their durability under environmental exposure, with reported responses to water ranging from satisfactory levels of adhesion (*ITSR* > 90%) [19] to reductions in indirect tensile strength due to failures at the aggregate–binder interface [12,45]. In some systems, particle agglomeration and thermal instability have been proposed as possible contributing factors [12].

Although the incorporation of bio-oils from biomass into conventional bituminous binders has been widely investigated, their behaviour is influenced by the type of biomass, bio-oil pre-treatment and the properties of the reference bitumen. Consequently, findings obtained for a particular bio-oil cannot necessarily be generalised to bio-oils derived from different biomass feedstocks or subjected to different processing conditions. Furthermore, fewer studies have examined the effects of specific pre-treatment conditions on the resulting bio-oil properties.

Within this context, this research focuses on the laboratory pre-treatment of olive-biomass-derived pyrolysis bio-oil and evaluates the resulting bio-binders and asphalt mixtures. The specific contribution lies in the experimental comparison of different pre-treatment conditions and the subsequent assessment of the selected bio-binder formulation and the corresponding bituminous mixture. The behaviour of the bio-binders in comparison with conventional reference binders (35/50 and 50/70) is evaluated. The mixtures are characterised in terms of volumetric properties, moisture susceptibility, and cracking resistance.

The specific objectives of this study are:

- To identify suitable laboratory pre-treatment conditions for bio-oil derived from the pyrolysis of residual olive biomass.
- To analyse the effects of incorporating bio-oil as a partial substitute for conventional bitumen on the properties of the bio-binder.
- To evaluate different bio-oil replacement levels for producing bio-binders and select a suitable formulation based on the volumetric and mechanical properties of BBTM 11B bituminous mixtures.

## 2. Materials and Methods

### 2.1. Materials

#### 2.1.1. Bio-Oil

The bio-oil used in this study was obtained from residual biomass from olive cultivation processed in an industrial biomass pyrolysis facility located in southern Spain. According to the technical information provided by the supplier, the pyrolysis process operated at temperatures ranging from 600 to 630 °C, with a reported heating rate of 20–30 °C/min and a residence time of approximately 30 min at the maximum temperature.

The bio-oil from pyrolysis exhibited a density of 1.213 g/cm^3^ and an initial mass loss of 53.2%, as measured using a thermogravimetric moisture analyser. As shown in Figure 1, the bio-oil was dark brown to black and had a viscous appearance. The characteristics of pyrolysis bio-oils are strongly influenced by the biomass feedstock and the pyrolysis conditions. Consequently, the results obtained in this study are specifically associated with the bio-oil from olive biomass investigated, and their applicability to bio-oils from other feedstocks requires further verification. The characteristics of bio-oils and their interactions with petroleum-derived bitumen have been discussed in previous studies [46].

#### 2.1.2. Bituminous Binders

For the production of the binders, conventional PEN 35/50 and PEN 50/70 bitumens were selected. These are standardised in accordance with EN 12591 [64] and are widely used in Europe (particularly in regions with temperate and warm climates) for the production of bituminous mixtures for wearing courses. In the first stage of the process, the PEN 35/50 binder was used with the addition of bio-oil; subsequently, once a formulation had been selected, the procedure was replicated using the PEN 50/70 binder. The selection of both binders was intended to assess whether bio-oil acts primarily as a softening or rejuvenating agent, as well as to evaluate whether its incorporation affects binder consistency, in accordance with the specifications presented in Table 1.

The requirements for conventional 35/50 and 50/70 bitumens were established according to EN 12591 [64] and Article 211 of the Spanish PG-3 specifications [65].

**Table 1:** Properties of 35/50 and 50/70 binders in accordance with European and Spanish regulations.

Properties	Unit	Standard	35/50	50/70
Penetration at 25 °C	(0.1 mm)	EN 1426 [66]	35–50	50–70
Softening point	°C	EN 1427 [67]	50–58	46–54
Ageing resistance at 163 °C (RTFOT)				
Mass change, max.	%	EN 12607-1 [68]	0.5	0.5
Retained penetration, min.	%	EN 1426 [66]	53	53
Increase in softening point, max.	°C	EN 1427 [67]	11	10

#### 2.1.3. Bituminous Mixture

The bituminous mixture selected was BBTM 11B. This is a gap-graded mix with a maximum aggregate size of 11 mm, which is laid as a wearing course in thin layers. Within the road pavement structure, this mixture forms the bituminous layer that must meet the most stringent requirements, as it is in direct contact with vehicle tyres and atmospheric conditions, and also because it has a gap-graded structure and is placed in thin wearing courses.

Porphyry coarse aggregate, limestone fine aggregate, and a calcium carbonate (CaCO_3_) filler were used, with a binder content of 4.75% by weight of the mix. The binder content selected corresponds to the minimum reference binder content specified for BBTM 11B bituminous mixtures in Article 543 of the Spanish General Technical Specifications for Road and Bridge Works (PG-3). This dosage was selected to assess the mixtures at the adopted minimum reference binder content, at which performance was expected to be most critical. The aggregate gradation is shown in Figure 2.

### 2.2. Methods

#### 2.2.1. Pre-Treatment of Bio-Oil

In general, bio-oil contains a high proportion of water, which originates both from the initial moisture content of the raw material and from the dehydration reactions that occur during pyrolysis, with the proportion varying depending on the type of biomass used and the processing conditions. This water content must be removed by means of a thermochemical dehydration process prior to the blending stage [13].

The bio-oil was pre-treated using a thermal process, varying the temperature, stirring speed (rpm) and treatment duration. For this purpose, a Silverson L5 high-shear laboratory mixer was used, fitted with a high-shear square-hole mesh disintegrator head (Figure 3). The mixer can operate at speeds of up to 10,000 rpm. The reduction in measured mass loss observed during thermal pre-treatment is consistent with the combined evaporation of water and the release of light volatile compounds from the bio-oil [24,69]. This joint release during the initial heating phase is supported by thermal characterisations reported in the literature [70].

To ensure precise control of the experimental temperature conditions, the equipment was connected to a thermal system comprising a container filled with silicone oil and a digital temperature controller, which enabled the samples to be maintained at the set temperature and ensured precise control of the stirring and heating conditions. The temperature of the samples was additionally monitored using a separate thermometer, ensuring thermal uniformity throughout the process [71].

Eight bio-oil pre-treatment procedures were initially investigated by combining different pre-treatment durations, stirring speeds, and heating temperatures. These procedures were identified as BT1 and B2-B8, as shown in Table 2. The codes B2-B8 refer to experimental series conducted at the same temperature and stirring speeds during the pre-treatment, with measurements obtained at different treatment durations. In contrast, BT1 corresponds to a sequential treatment consisting of two stages, the first of 0.5 h at 100 °C and 500 rpm, and the second of 1.5 h at 110 °C and 2000 rpm. Among the different pre-treatment procedures studied, the BT1 pre-treatment was adapted from a previous study [72] to evaluate the response of the treated bio-oil. The series studied represent different experimental conditions rather than independent repetitions of the same treatment. The pre-treatment procedures were evaluated by considering the evolution of mass loss, treatment duration, and visible changes in the bio-oil. Based on these observations, selected conditions were subsequently used to prepare bio-binders.

#### 2.2.2. Preparation of the Bio-Binder

During bio-binder preparation, both components were preheated separately in an oven—the bio-oil at 100 °C and the asphalt binder at 130 °C—in order to reduce their viscosity, facilitate handling and ensure uniform thermal conditions during mixing.

Subsequently, both materials were introduced into a high-shear laboratory mixer fitted with a general-purpose disintegrator head. Mixing was carried out at 135 °C, at a constant stirring speed of 5000 rpm for 15 min. This speed was selected to provide sufficient shear forces to promote adequate dispersion of the bio-oil within the bituminous binder matrix. The operating conditions were established on the basis of previous studies which identified these parameters as suitable for achieving a homogeneous mixture [7,35,46].

After mixing, each bio-binder was transferred to an oven for 15 min to reach 150 ± 5 °C, before bituminous mixture production. The temperature was monitored using a digital thermometer. Once the bio-binder reached the target temperature, production of the asphalt mixture specimens began. All bio-binders were subjected to the same conditions to ensure the reproducibility of the study. The purpose of this temperature increase was to impart the required flow properties and operational viscosity before preparing the hot-mix asphalt, in accordance with the protocols reported in the literature [19]. Figure 4 presents a flowchart of this procedure, from the initial preheating of the components to the thermal stabilisation of the final bio-binder in the oven.

#### 2.2.3. Selection of Bio-Binders for the Production of Bituminous Mixtures

The bio-binder formulations were initially prepared using 35/50 bitumen as the reference binder. Based on the literature review [4,18], four bio-oil replacement levels and a control were selected for the experimental programme: 0%, 2.5%, 5%, 10%, and 20% of the total binder mass. These percentages refer to the total mass of the binder and not the total mass of the asphalt mixture. Accordingly, a 10% replacement level corresponds to 10% bio-oil and 90% conventional binder by mass within the binder content. These dosages were selected to investigate the effect of increasing bio-oil content on the properties of the resulting bio-binder. A 35/50 bitumen was used as the reference for comparison.

After selecting the bio-oil replacement percentage based on the results obtained, the study was repeated using a 50/70 penetration-grade binder, with the aim of comparing the influence of the bitumen’s penetration on both the behaviour of the resulting bio-binder and that of the bituminous mixture.

Table 3 summarises the different bio-binder formulations prepared using the selected bio-oil pre-treatment conditions. To distinguish the pre-treatment procedure from the resulting bio-binders, the codes BW1–BW6 were assigned to specific combinations of temperature, stirring speed, and treatment duration selected previously (Table 2). Specifically, BW1, BW2, and BW3 correspond to procedure B3 at 5, 7, and 9.5 h, respectively; BW4 corresponds to B6 at 2.5 h; BW5 to B4 at 8 h; and BW6 to B2 at 15 h. Treatments B5, B7, and B8 were excluded from subsequent bio-binder preparation because visible physical alterations were observed in the bio-oil. The formulation codes indicate the percentage of bio-oil incorporated into the conventional bitumen followed by the corresponding pre-treatment code. For example, 10%BW1 represents a bio-binder containing 10% bio-oil pre-treated under BW1 conditions. The binder containing 10% untreated bio-oil was identified as 10%B.SP. Finally, the BT1 treatment was evaluated using 10% bio-oil with both 35/50 and 50/70 bitumens.

### 2.3. Laboratory Testing

This section describes the tests carried out on bio-oils, bio-binders and bituminous mixtures produced using bio-binders.

#### 2.3.1. Determination of Thermogravimetric Mass Loss in Bio-Oil

The mass loss of the bio-oils was determined using the thermobalance evaporation method, in accordance with standard EN 16849 [73]. A thermogravimetric moisture analyser consisting of a precision balance and a heating chamber was used. This method measured the reduction in sample mass during heating and was used to evaluate the changes occurring in the bio-oil following the different thermal pre-treatment procedures considered.

Samples weighing more than 4 g were analysed at a set temperature of 120 °C. The measured temperature was 119 °C, within the specified tolerance of ±3 °C. The instrument automatically recorded the mass change. A bitumen sample was also analysed under the same conditions, yielding a reported mass loss of 0%. The results were used to assess the mass loss using the instrumental method employed (Figure 5).

The mass loss (*ML*), expressed as a percentage by mass, was determined using Equation (1):

(1) $$ML=\frac{m_{0}-m_{f}}{m_{0}}\cdot 100$$

where *m*_0_ corresponds to the initial mass of the sample and *m*_f_ is the final mass at the end of the heating programme.

During the test, the samples were evenly distributed in a thin layer on an absorbent filter placed on the weighing pan, in order to promote uniform evaporation. The test duration ranged from 20 to 115 min, depending on sample characteristics and the measured mass-loss behaviour.

It should be noted that the measured mass loss may result from the evaporation of both water and volatile organic compounds present in the bio-oil. Therefore, the values obtained should not be interpreted as direct measurements of water content, but rather as indicators of the volatile fraction released under the testing conditions.

This approach enables a comparative assessment of the effects of thermal pre-treatment on the bio-oil, although it does not allow the individual contributions of water and organic volatiles to be quantified.

#### 2.3.2. Penetration and Softening Point Tests on Binders

The consistency of the conventional bitumens (35/50 and 50/70) and the bio-binders was characterised by measuring penetration at 25 °C in accordance with EN 1426 [66] and softening point in accordance with EN 1427 [67]. The properties of the reference bitumens are presented in Table 4, while the bio-binders correspond to the formulations described in the previous section. For each binder formulation, at least three penetration measurements and two softening point determinations were performed.

In addition, each sample was subjected to short-term ageing in a rotary oven (RTFOT) in accordance with standard EN 12607-1 [68].

The penetration test was carried out under controlled conditions of load and application time, measuring the penetration depth of a standardised needle as an indicator of the binder’s consistency at a temperature of 25 °C.

The softening point was determined using the Ring-and-Ball method, by recording the temperature at which the metal ball descended the standard distance as a result of the softening of the binder.

#### 2.3.3. Rolling Thin-Film Oven Test (RTFOT)

Resistance to ageing caused by heat and air was assessed using the rolling thin-film oven test (RTFOT), which simulates the short-term ageing experienced by the binder during the mixing and placement processes. The samples were subjected to a temperature of 163 °C for 75 min, with an air flow rate of (4.0 ± 0.2) L/min (Figure 6). Subsequently, the aged binders were characterised using penetration and softening point tests, in order to assess changes in their physical properties and their susceptibility to ageing.

#### 2.3.4. Tests on Bituminous Mixtures Produced Using Bitumens and Bio-Binders

The volumetric and mechanical properties of the BBTM 11B mixtures prepared using the reference binders and the bio-binders obtained were determined, as described below.

- Air void content

Test specimens of BBTM 11B-type bituminous mixtures, formulated with the various bio-binders, were produced and compacted using a Marshall impact compactor, applying 50 blows per face in accordance with the EN 12697-30 standard [74]. This procedure yielded standardised cylindrical specimens with a nominal diameter of 101.6 mm and an approximate height of 63.5 mm.

Once the specimens had been conditioned, the air void content was determined in accordance with the protocol set out in standard EN 12697-8 [75]. This volumetric parameter was calculated mathematically from the bulk density of the compacted test specimens in accordance with standard EN 12697-6 [76] and the theoretical maximum density of the mixture in its loose state in accordance with EN 12697-5 [77].

The air void content was measured for all specimens subsequently used in the indirect tensile strength and Fénix tests.

- Indirect tensile strength test

The indirect tensile strength of the bituminous mixtures was assessed using the indirect tensile test, in accordance with the guidelines set out in standard EN 12697-23 [78]. This test enables the indirect tensile strength of the mixture to be determined from the maximum load applied to the specimen at the point of failure and from its geometric dimensions (Figure 7). The value of the indirect tensile strength (*ITS*) is calculated using Equation (2).

(2) $$ITS=\frac{2P}{\pi DH}$$

where *ITS* is the indirect tensile strength (MPa), *P* is the maximum load (N), *D* is the diameter of the test specimen (mm) and *H* is the height of the test specimen (mm).

The water sensitivity of the mixtures was assessed in accordance with standard EN 12697-12 [79]. Six test specimens were produced for each mixture, which were divided into two groups of three specimens. The first group was kept under dry conditions (*ITS_d_*), whilst the second group was subjected to moisture conditioning by vacuum saturation at an absolute pressure of 6.7 kPa for 30 min, followed by immersion in water at 40 °C for 72 h (*ITS_w_*). The indirect tensile test was carried out at 15 °C.

The retained strength, expressed as the indirect tensile strength ratio (*ITSR*), was calculated as the ratio of the average strength of the conditioned specimens to the average strength of the dry specimens, as shown in Equation (3).

(3) $$ITSR=\frac{{ITS}_{w}}{{ITS}_{d}}\times 100$$

where (*ITS_w_*) is the average strength of the specimens conditioned by immersion in water and (*ITS_d_*) is the average strength of the specimens tested in dry conditions.

For each mixture, six specimens were tested: three under dry conditions and three under wet conditions. The *ITSR* was calculated as the ratio of the mean direct tensile strength of the wet specimens to that of the dry specimens, expressed as a percentage. Therefore, a single *ITSR* value was obtained for each mixture formulation from the two group means. Unlike the individual *ITS* measurements, the *ITSR* was not determined separately for each specimen, and no error bars are reported for this calculated parameter.

- Fénix test

The Fénix test is used to assess the cracking resistance of bituminous mixtures, in accordance with standard NLT-383. This test involves applying a tensile load to a semi-cylindrical specimen with a central notch, in order to induce and control the cracking process. The load is transmitted via metal plates attached to the specimen and secured to the test machine’s anchors (Figure 8).

The Fénix test was carried out under displacement control at a constant speed of 1 mm/min. The test specimens were evaluated at temperatures of 20, 5 and −5 °C. The test was performed using three replicates for each binder and testing temperature considered. During the test, the applied load was continuously recorded as a function of displacement, yielding the corresponding load–displacement curve for each test specimen. From these curves, the mechanical parameters used to evaluate the fracture behaviour of the bituminous mixtures were determined: the tensile strength (*RT*) and the ductility (*DT*) of the mixture.

The *RT* parameter is the maximum stress obtained in the test (Equation (4)).

(4) $$RT=1000*\frac{F_{max}}{S}$$

where *F_max_* is the maximum load (N) and *S* is the fracture area of the specimen (mm^2^).

The *DT* parameter is the ductility or tenacity displacement (mm), and is measured as the displacement from the point at which the maximum load is reached (*d_Fmax_*) until the load is reduced to half its maximum value during the fracture phase (*d*_50*pm*_), as shown in Equation (5).

(5) $$DT=d_{50pm}-d_{Fmax}$$

This test has been widely used on various types of mix, both for the assessment of laboratory-produced test specimens and for quality control of field samples [81,82,83].

The results of the different tests are expressed as mean values and standard deviations. The technical assessment of the BBTM 11B asphalt mixtures was based on the Spanish General Technical Specifications for Road and Bridge Works (PG-3), Article 543 [65]. The acceptance criteria considered in this study were an air void content between 12% and 18%, and a minimum indirect tensile strength ratio (*ITSR*) of 90%.

## 3. Results

### 3.1. Pre-Treatment of Bio-Oil

The thermal pre-treatment was applied to the eight experimental series. Pre-treatment changed the measured thermogravimetric mass loss and the macroscopic appearance of the bio-oil. Bubbling and expansion were observed as the material was heated. Subsequently, at around 2 h into the treatment, the system temperature stabilised in accordance with the parameters programmed into the equipment. During this process, periodic samples were taken to quantify the reduction in mass loss. These samples were stored for subsequent analysis.

Figure 9 shows the evolution of the thermogravimetric mass loss measured after pre-treatment for each of the eight series as a function of thermal exposure time.

The pre-treatment response was evaluated using the eight experimental series shown in Figure 9, with the initial thermogravimetric mass loss of 53.2% as the reference (Table 5). Some experimental runs achieved estimated reductions of 90–95%, though these required processing times ranging from 6.5 to 9.5 h. Subsequent visual inspection revealed changes in appearance and the presence of solid particles in runs B5-T130-R6, B7-T120-R8, and B8-T130-R8, which were consequently excluded from further testing. The remaining samples were selected for the preparation of the bio-binder and the evaluation of its performance in bituminous mixtures, using PEN 35/50 bitumen as a reference.

The results are interpreted below:

- Low-severity series (BT1 and B2-T100-R4)

The BT1 reference sequential treatment, adapted from the literature [72], was carried out in two stages (100 °C at 500 rpm and 110 °C at 2000 rpm). Under these moderate conditions, the pre-treatment resulted in a 43% relative reduction in mass loss after 2 h of testing, without the visible alterations observed under more severe pre-treatment conditions. Although this reduction was lower than those obtained with several longer treatments, BT1 was subsequently evaluated based on the mechanical performance of the resulting bio-binder and asphalt mixture.

Meanwhile, the B2-T100-R4 series exhibited the slowest removal kinetics in the study, achieving a 72% relative reduction in measured mass loss after 15 h.

- Intermediate-severity series (B3, B4, B6, and B7)

The series treated under intermediate conditions showed markedly faster removal kinetics:

- Higher removal rate (B7-T120-R8 and B6-T120-R7). The reduction in measured mass loss increased dramatically in the B7-T120-R8 series, reaching 91% in 5 h, closely followed by B6-T120-R7, which achieved 95% in 6.5 h. The different treatment times suggest that the combined processing conditions influence the observed reduction in mass loss. However, it was observed that in the sample subjected to 8000 rpm (B7-T120-R8), black solid particles were present in the extracted samples, which also exhibited visible physical alterations.
- Moderate kinetics (B3-T110-R6 and B4-T120-R6). At a lower mass transfer rate, the B3-T110-R6 series took 9.5 h to reach a 94% relative reduction in measured mass loss, a behaviour similar to that of the B4-T120-R6 series, which took 8 h to achieve 90%. This suggests that an increase of 10 °C (whilst maintaining the same stirring speed) slightly accelerates the system’s kinetics, shortening the observed treatment duration under the investigated conditions, with no visible alterations reported for these samples.

- High-severity series (B5 and B8)

In the B5-T130-R6 and B8-T130-R8 series, a large reduction in measured mass loss was achieved from the very first hour of testing. Indeed, in the case of B8-T130-R8, the processing time was reduced to just 3 h to achieve 94% efficiency. However, it was found that operating under these processing conditions leads to visible physical alterations (superficial in B5-T130-R6 and severe in B8-T130-R8). These pre-treatments were excluded from subsequent bio-binder preparation because visible physical changes were considered undesirable.

The final selection was informed by mass-loss measurements, visual observations and subsequent binder and mixture performance.

### 3.2. Analysis of Bio-Oil

#### Mapping and Selection of the Operational Window

To summarise the results obtained from the eight experimental pre-treatment procedures, an operating-window map was developed based on stirring speed and temperature (Figure 10). According to the observed behaviour of the bio-oil under the investigated conditions, three distinct operating regions were identified:

- Zone 1 (Low severity): This zone encompasses the experimental conditions with the lowest severity identified in this study, corresponding to temperatures of 100–110 °C and agitation speeds ranging from 500 to 6000 rpm. Specifically, it includes series BT1, B2-T100-R4, and B3-T110-R6.
- Zone 2 (Moderate severity): Defined at 120 °C with stirring speeds of 6000 and 7000 rpm. This zone includes series B4 and B6. Under these conditions, the samples showed no macroscopic changes or visible residue formation. Consequently, these conditions were classified as moderate severity within the experimental scope.
- Zone 3 (High severity): Under the investigated processing conditions, samples B5, B7, and B8 exhibited physical alterations, including the formation of dark solid particles, some of which floated on the surface when immersed in water. These observations led to the exclusion of these treatments from further evaluation. Therefore, this region represents processing conditions associated with undesirable physical changes in the investigated bio-oil, rather than universal thresholds for thermal or mechanical degradation.

### 3.3. Characterisation of the Bio-Binder

#### 3.3.1. Penetration and Softening Point Tests

The results for penetration (EN 1426 [66]) and softening point (EN 1427 [67]) are presented for the bio-binders designed using the 35/50 base binder and bio-oil additions of 2.5%, 5%, 10%, and 20%, with different mass loss values (Figure 11). In terms of regulatory compliance, most bio-binders met the evaluated penetration and softening point criteria, except samples 10%BW1 and 5%BW2, which did not achieve the minimum penetration value, and sample 10%BW4, which fell below the lower limit for the softening point.

However, the penetration and softening point results exhibited a non-linear relationship with partial replacement of bitumen with of bio-oil, as the physical consistency parameters (penetration and softening point) were governed by the mass loss (ML) of the pre-treated bio-oil. The bio-binders are categorised into two regimes according to their residual mass loss.

- Regime I (ML ≤ 8%). In mixtures formulated with bio-oil with low residual mass loss (5.0%BW2, 10%BW2, 10%BW3, 20%BW3, and 10%BW5), the softening effect of the bio-oil predominates at higher replacement levels. An increasing trend in penetration is observed at dosages of 10% and above, reaching values up to 40.7 × 0.1 mm in the BW3 mixtures, whereas the softening point remains within a stable range between 51.3 °C and 54.8 °C, well above the 50 °C reference line.
- Regime II (ML > 8%). In mixtures with higher residual mass loss (10%B.SP, 10%BT1 + 35/50, BW1, BW4, and 20%BW6), the consistency parameters do not maintain a linear trend. On the one hand, the BW1 mixtures (2.5%, 5.0%, and 10%) maintain a practically constant penetration close to the base specification level. On the other hand, specific deviations are observed; for instance, in sample 10%BT1 + 35/50, an increase in penetration to 40.7 × 0.1 mm coexists with an elevated softening point of 54 °C, while in sample 10%BW4, the softening point drops to 49 °C, falling below the reference line. The penetration of the formulation containing untreated bio-oil was 36.7 dmm; the cause of this behaviour cannot be determined from the available tests [84].

Previous studies have highlighted the relevance of pre-treatment for improving handling and performance [18,41,85,86,87]. These studies have reported that bio-oil incorporation may increase penetration and reduce the softening point of the bio-binder compared with the conventional binder. Although an increase in penetration was also observed in this research, the softening point did not always decrease. This difference highlights the influence of bio-oil origin, bio-oil content, and bio-binder preparation on consistency. In particular, the use of bio-oil from the pyrolysis of olive biomass and the pre-treatments may contribute to the differences observed relative to previous studies. Nevertheless, direct comparison between studies is limited by differences in biomass composition and reference binder properties, among other factors.

#### 3.3.2. Susceptibility to Short-Term Ageing (RTFOT)

After the bio-binders were subjected to short-term ageing using the rolling thin-film oven test (RTFOT) in accordance with standard EN 12607-1 [68], the binders manufactured using bio-oil exhibited penetration values close to the standard and softening points ranging from 56.3 °C to 63.0 °C.

When assessing the specific control parameters required by the EN 12591 [64] specification standard to evaluate susceptibility to ageing, favourable behaviour was first observed in the consistency properties: both retained penetration (percentage relative to the initial state) and the increase in the softening point (temperature change in °C) complied with the acceptable limits in all the combinations studied (Figure 12).

The mass change measured after RTFOT ageing provides an additional indicator of the thermal response of the bio-binders. As shown in Table 6, most bio-binders exhibited mass losses within the specified limit of 0.5%. However, four bio-binders (20%BW3, 10%BW4, 10%BW5, and 20%BW6) exceeded this threshold. These bio-binders were classified as non-compliant with the evaluated mass loss criterion.

The differences observed among bio-binders suggest that the response to short-term ageing depends on the bio-oil replacement percentage and pre-treatment conditions. The measured mass losses may be partly associated with the evaporation of volatile components. Nevertheless, other physicochemical changes may also occur during short-term ageing, and the available measurements do not allow these mechanisms to be distinguished.

With regard to the mass loss of the formulated bio-binders, formulations BW1 to BW6 exhibited low values, below 1% (between 0.0% and 0.6%), similar to those published in a previous study [14].

In contrast, the bio-binder produced from unprocessed bio-oil had a mass loss of 2.2%, whilst the 10%BT1 + 35/50 formulation reached a value of 2.1%. These results demonstrate greater moisture retention in the bio-binders formulated with unprocessed bio-oil and using a short-duration process, compared with those prepared from pre-treated bio-oils.

### 3.4. Results Obtained for Bituminous Mixtures Containing Bio-Binder

Fourteen series of BBTM 11 B bituminous mixtures were produced using the 35/50 binder and the resulting bio-binders. The properties assessed for each of these are set out below, and their compliance with the technical specifications is analysed.

#### 3.4.1. Volumetric Properties

The volumetric properties obtained in accordance with EN 12697-8 [75] showed that the incorporation of bio-oil generally increased the air void content of BBTM 11B mixtures compared with the reference mixture. However, no proportional relationship was observed between bio-oil content and air void percentage, suggesting that the volumetric response may also depend on binder consistency, bio-oil pre-treatment conditions, and aggregate rearrangement during compaction.

As shown in Figure 13, the reference mixture (35/50 binder) and those containing 10%B.SP, 10%BT1 + 35/50, 2.5%BW1, and 20%BW6 exhibited air void contents within the specified range of 12–18%. The mixture containing 5.0%BW1 approached the upper limit, whereas the remaining eight mixtures (10%BW1, 5%BW2, 10%BW2, 10%BW3, 20%BW3, 5%BW4, 10%BW4, and 10%BW5) exceeded the maximum permitted value of 18%.

#### 3.4.2. Mechanical Properties (Water Sensitivity) of Mixtures Using 35/50 Bitumen

Figure 14 presents the mean indirect tensile strength (*ITS*) values obtained under dry and wet conditions for the control mixture (35/50) and the mixtures prepared with bio-binders. In general, incorporating bio-oil into the binder resulted in lower tensile strength values compared with the reference mixture.

Moisture susceptibility was assessed using the indirect tensile strength ratio (*ITSR*), calculated from the mean *ITS* values obtained for the wet and dry specimens. As shown in Figure 15, most mixtures satisfied the adopted *ITSR* ≥ 90% criterion, except 20%BW3, 10%BW5, and 20%BW6. Since a single *ITSR* value was calculated for each mixture from the mean *ITS* values of three wet and three dry specimens, no error bars are presented for this parameter.

### 3.5. Overall Technical Feasibility

To evaluate the overall technical feasibility of the analysed bio-binders, a qualitative and quantitative compliance matrix was constructed (Table 7), comparing the physical properties of the bio-binders before and after RTFOT ageing, as well as the volumetric and mechanical properties of the bituminous mixtures, against the Spanish specification limits for the 35/50 reference binder and BBTM 11B-type mixtures. Based on this comprehensive analysis, the following was observed:

- Bio-binder properties before ageing: the penetration and softening point tests generally demonstrated compliance with the technical specifications. The only exceptions were samples 10%BW4 (softening point) and 5.0%BW2 and 10%BW1 (penetration).
- Bio-binder properties after ageing (RTFOT): retained penetration and the increase in softening point met the requirements across all formulations. However, regarding mass change, samples 20%BW3, 10%BW4, 10%BW5, and 20%BW6 exceeded the maximum allowable value of 0.5%.
- Volumetric properties of the bituminous mixture with bio-binder, air void content: formulations 10%BW2, 10%BW3, and 20%BW3 exceeded the upper limit of 18%, while mixtures 10%BW1, 5.0%BW2, 5.0%BW4, 10%BW4, and 10%BW5 also were above the permitted range although they did so to a lesser extent. This excessive void content may be associated with differences in compactability, which was not directly measured.
- Water sensitivity of the bituminous mixture with bio-binder (*ITSR*): high bio-oil content (20%BW3 and 20%BW6) resulted in low indirect tensile strength under wet conditions, reducing the *ITSR* to 67% and 31%, respectively. Similarly, sample 10%BW5 (87%) failed to meet the minimum threshold of 90%, although its value was closer to the required limit than those of mixtures containing binders with 20% bio-oil.

As a result of the overall analysis, four modified mixtures (10%B.SP, 10%BT1 + 35/50, 2.5%BW1, and 5.0%BW1) met all seven evaluated technical criteria defined for the 35/50 base binder and the BBTM 11B mixture. These findings demonstrate the technical feasibility of incorporating up to 5% bio-oil under standard conditions (sample BW1) and up to 10% when a two-stage thermal pre-treatment is applied (100 °C and 110 °C, sample 10%BT1 + 35/50), while satisfying the laboratory acceptance criteria evaluated in this study.

The selection of the most suitable formulation was based on a combined assessment of technical performance, bio-oil replacement percentage, pre-treatment duration, and the physical condition of the treated material. Four formulations (10%B.SP, 10%BT1 + 35/50, 2.5%BW1, and 5.0%BW1) met all seven technical criteria evaluated in this study. However, the formulation containing untreated bio-oil (10%B.SP) was not selected due to its high initial mass loss, which could indicate the presence of a substantial volatile fraction. Although the 2.5%BW1 and 5.0%BW1 formulations also met the evaluated requirements, both required 5 h of pre-treatment. In contrast, the 10%BT1 + 35/50 formulation achieved satisfactory technical performance with only 2 h of pre-treatment, representing a 60% reduction in processing time compared with BW1. Furthermore, BT1 did not exhibit the visible degradation observed under more severe pre-treatment conditions. Therefore, 10%BT1 + 35/50 was selected as the most suitable bio-binder among those investigated, providing a favourable balance between bitumen replacement, pre-treatment duration, and compliance with the evaluated technical criteria. Although the BT1 pre-treatment reduced the duration of the procedure by 60% compared with BW1, no quantitative assessment of energy consumption, production costs or industrial-scale feasibility was performed. Therefore, the selected formulation should be considered the most suitable among the alternatives evaluated under laboratory conditions, rather than an economically optimal solution.

### 3.6. Evaluation Based on Selected Data and Replication Using the 50/70 Binder

After the 10%BT1 + 35/50 formulation had been selected, the same dosage was evaluated using a binder with greater penetration (50/70). The aim of this phase was to assess whether a similar response was obtained with a different base bitumen, by analysing the properties of the bio-binder as well as those of the BBTM 11B mixture. The comparative results are presented below.

#### 3.6.1. Penetration Test and Softening Point Before and After RTFOT Ageing

The results for the properties of the 35/50 and 50/70 binders, and the bio-binders obtained by replacing 10% of the binder with bio-oil, are presented in Table 8.

Table 8 presents the results of the physical characterisation of the binders in their initial state and after short-term ageing (RTFOT). Compared with the binders in their initial state, the incorporation of 10% bio-oil (BT1) had a softening effect on both standard binders, although to a varying degree depending on the penetration of the base binder. In the softer binder (50/70), the addition of bio-oil produced a marked increase in penetration from 50.7 to 58.0 dmm, accompanied by a rise in the softening point from 51.0 °C to 54.0 °C.

Following short-term accelerated ageing (RTFOT), both bio-binder series met the evaluated retained-penetration and softening point change criteria. The bio-binders exhibited smaller changes in softening point than the reference binders under the applied RTFOT procedure. Both formulations satisfied the adopted RTFOT mass-change limit of 0.5% under the investigated conditions.

#### 3.6.2. Volumetric Properties of Mixtures Prepared Using Bio-Binders

Figure 16 shows the void content for the four mixtures evaluated (two reference mixtures and two mixtures produced with 10%BT1), considering a specified minimum limit of 12% and a maximum limit of 18%. As can be seen, all mixtures fell within the specified range.

The mixtures containing 10%BT1 showed a slight increase in the void content compared with those of the reference mixtures. This effect was most noticeable in the mixture with the stiffer 35/50 reference binder, where the void content rose from 15.8% to 17.8% (approaching the permitted limit). In contrast, with the 50/70 binder, the variation was practically negligible, rising from 15.9% in the reference sample to 16.4% in the sample containing the bio-binder. This behaviour demonstrates that the incorporation of 10%BT1 maintains an adequate volumetric density with both types of binder.

#### 3.6.3. Mechanical Properties (Water Sensitivity) of the Mixtures with 50/70 Bitumen

The results for indirect tensile strength (*ITS*) under dry and wet conditions are presented in Figure 17a, while Figure 17b shows the indirect tensile strength ratio (*ITSR*) to assess the water sensitivity of the four mixtures.

The mixtures modified with 10%BT1 showed a slight decrease in the absolute *ITS* values (both dry and wet) compared with their respective 35/50 and 50/70 control mixtures. For the 35/50 binder, the dry *ITS* fell from 1.7 MPa to 1.4 MPa, whilst for the 50/70 binder it decreased from 1.3 MPa to 1.1 MPa. This reduction in maximum mechanical strength is consistent with the increase in penetration in both cases.

However, when assessing water sensitivity (Figure 17b), the results show an indirect tensile strength ratio above 90%, meeting the requirements for BBTM 11B-type bituminous mixtures. Some *ITSR* values approached or exceeded 100%. This may reflect measurement variability or changes associated with conditioning. Previous research has suggested that conditioning specimens in water at 40 °C for 72 h could cause slight ageing of the mixture, increasing its stiffness [88]. These results indicate that the mixtures with 10%BT1 exhibited satisfactory performance in terms of moisture susceptibility with both bitumens, despite the reduction in indirect tensile strength values compared to the reference mixture. Both mixtures with bio-binders showed a high retained strength exceeding the minimum requirement, indicating satisfactory performance in the moisture susceptibility test.

This behaviour demonstrates that the 10%BT1 bio-oil does not compromise the mechanical properties of the mixture, but rather preserves interfacial adhesion in the presence of moisture [88].

#### 3.6.4. Resistance to Cracking (Fénix Test)

The cracking resistance of the asphalt mixtures was evaluated using the Fénix test at −5, 5, and 20 °C. The analysis focuses on the tensile strength (*RT*) and ductility (*DT*) parameters, as shown in the Fénix diagram (Figure 18). The corresponding mean values and standard deviations are presented in Table 9. For each combination of bitumen and test temperature, three replicate specimens were tested.

For mixtures prepared with 35/50 bitumen, the incorporation of 10%BT1 resulted in relatively similar *RT* and *DT* values at −5 and 5 °C. At 20 °C, the mixture with bio-binder exhibited reductions of approximately 12% in tensile strength and 14% in ductility compared with the reference mixture. For mixtures with 50/70 bitumen, *RT* values remained relatively close to those of the reference mixture for the different test temperatures evaluated. Nevertheless, the mixtures with bio-binder exhibited changes in ductility, indicating that bio-oil incorporation may influence the deformation response during the fracture process.

In general, the addition of 10% bio-oil maintained a satisfactory tensile strength response in the Fénix test, although ductility decreased under certain conditions.

## 4. Conclusions

This study evaluated the technical feasibility of partially replacing conventional 35/50 and 50/70 penetration-grade bitumens with pre-treated bio-oil from the pyrolysis of olive biomass. The analysis included the pre-treatment of bio-oil, bio-binder characterisation before and after RTFOT ageing, and the volumetric and mechanical properties of BBTM 11B bituminous mixtures. The main conclusions are as follows:

- The pre-treatment results showed that the response of bio-oil depended on the combined effects of temperature, stirring speed, and duration. Visible physical alterations were observed under certain high-severity processing conditions, although chemical degradation could not be confirmed. The BT1 pre-treatment, consisting of 100 °C at 500 rpm for 30 min followed by 110 °C at 2000 rpm for 90 min, was selected among the studied conditions because it provided suitable processing behaviour within a total duration of 2 h.
- The evaluation of bio-binder and bituminous mixture properties identified four formulations that satisfied all the technical criteria considered: 10%B.SP, 10%BT1 + 35/50, 2.5%BW1, and 5.0%BW1. Among the investigated pre-treated bio-oils, 10%BT1 was selected because it combined a 10% replacement of conventional bitumen with a shorter pre-treatment duration of 2 h, compared with the 5 h required by BW1. Therefore, the 10%BT1 formulation was considered the most suitable among those investigated, providing a favourable balance between bio-oil replacement percentage, pre-treatment duration, and technical performance. Nevertheless, further assessment of energy consumption, production costs, and industrial-scale processing requirements would be necessary to establish its economic feasibility.
- The incorporation of 10%BT1 increased penetration from 50.7 to 58.0 dmm for the 50/70 binder, while the softening point increased from 51.0 °C to 54.0 °C. For the 35/50 binder, penetration increased from 38.5 to 40.7 dmm, while the softening point remained at 54.0 °C. These results indicate that the influence of bio-oil on binder consistency depends on the characteristics of the reference bitumen.
- The 10%BT1 bio-binders satisfied the evaluated RTFOT short-term ageing criteria for retained penetration, softening point, and mass change. Mean RTFOT mass losses of 0.26% and 0.19% were obtained for the bio-binders containing 35/50 and 50/70 bitumens, respectively, remaining below the regulatory limit of 0.5%.
- The BBTM 11B mixtures incorporating 10%BT1 met the void content requirements within the specified range of 12% to 18% and the minimum *ITSR* requirement of 90%. Nevertheless, the mixtures with bio-binder exhibited lower indirect tensile strength than their corresponding reference mixtures. Therefore, the *ITSR* results indicate satisfactory performance in the moisture susceptibility test, rather than an overall improvement in mechanical performance.
- The Fénix test results showed that the incorporation of 10%BT1 bio-oil produced limited changes in tensile strength for the three temperatures evaluated, although reductions in ductility were observed for certain binder and temperature combinations. These findings suggest that the incorporation of 10%BT1 in asphalt mixtures maintains a satisfactory mechanical response in terms of tensile strength.

From a sustainability perspective, the incorporation of bio-oil from the pyrolysis of olive biomass offers a potential pathway for reducing the consumption of petroleum-based bitumens and promoting the valorisation of biomass-derived resources. This study demonstrates at laboratory scale the technical feasibility of replacing 10% of the total binder mass with bio-oil in terms of bio-binder consistency, short-term ageing behaviour, volumetric properties of mixtures, moisture susceptibility, and cracking resistance.

Nevertheless, the experimental programme did not include long-term ageing, fatigue or permanent deformation testing, storage stability assessment, or field validation. Further research is therefore required to evaluate the long-term performance of mixtures with bio-binder.

## Figures and Tables

**Figure 1:**
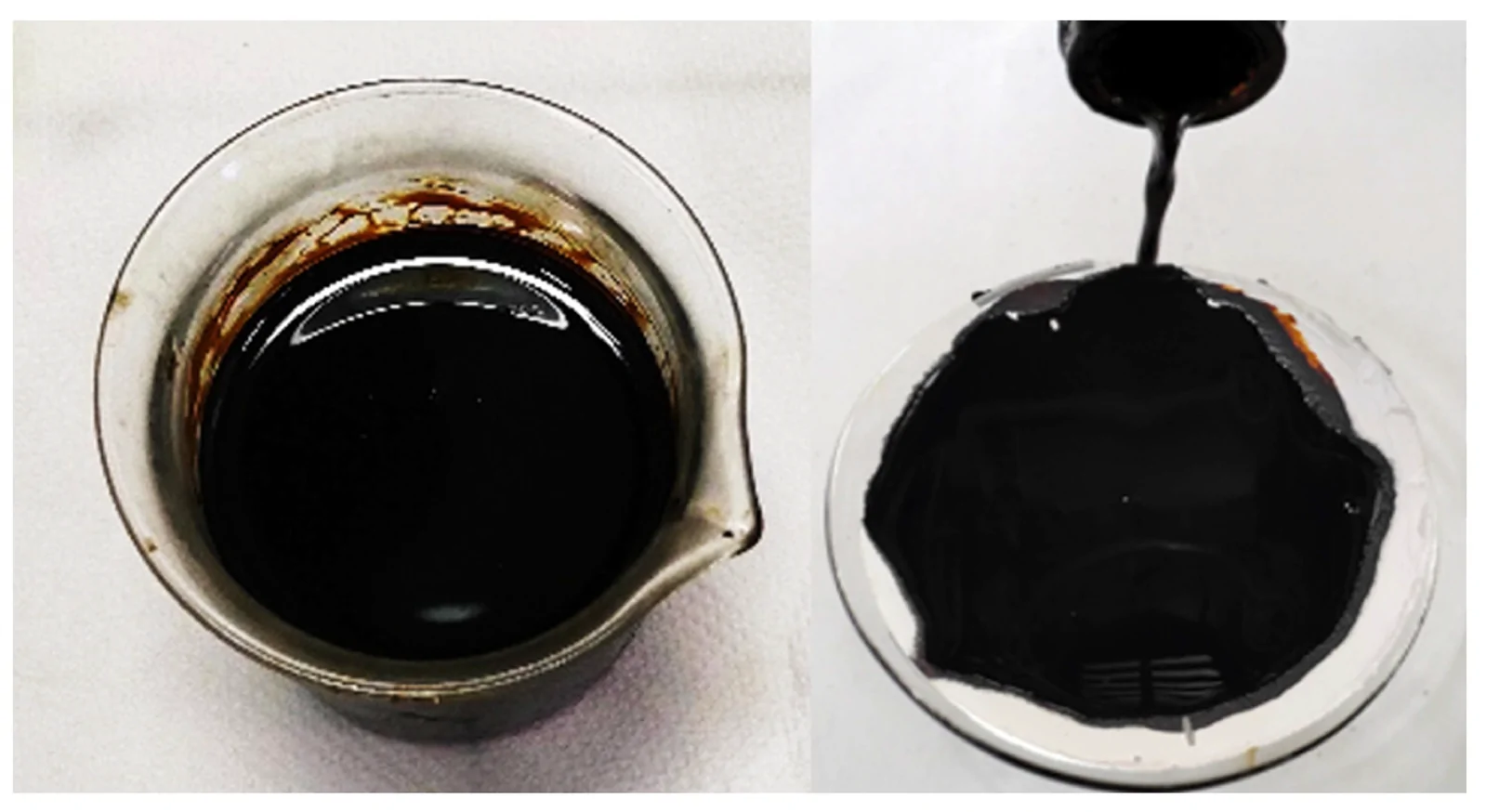
Pyrolysis bio-oil used in this study.

**Figure 2:**
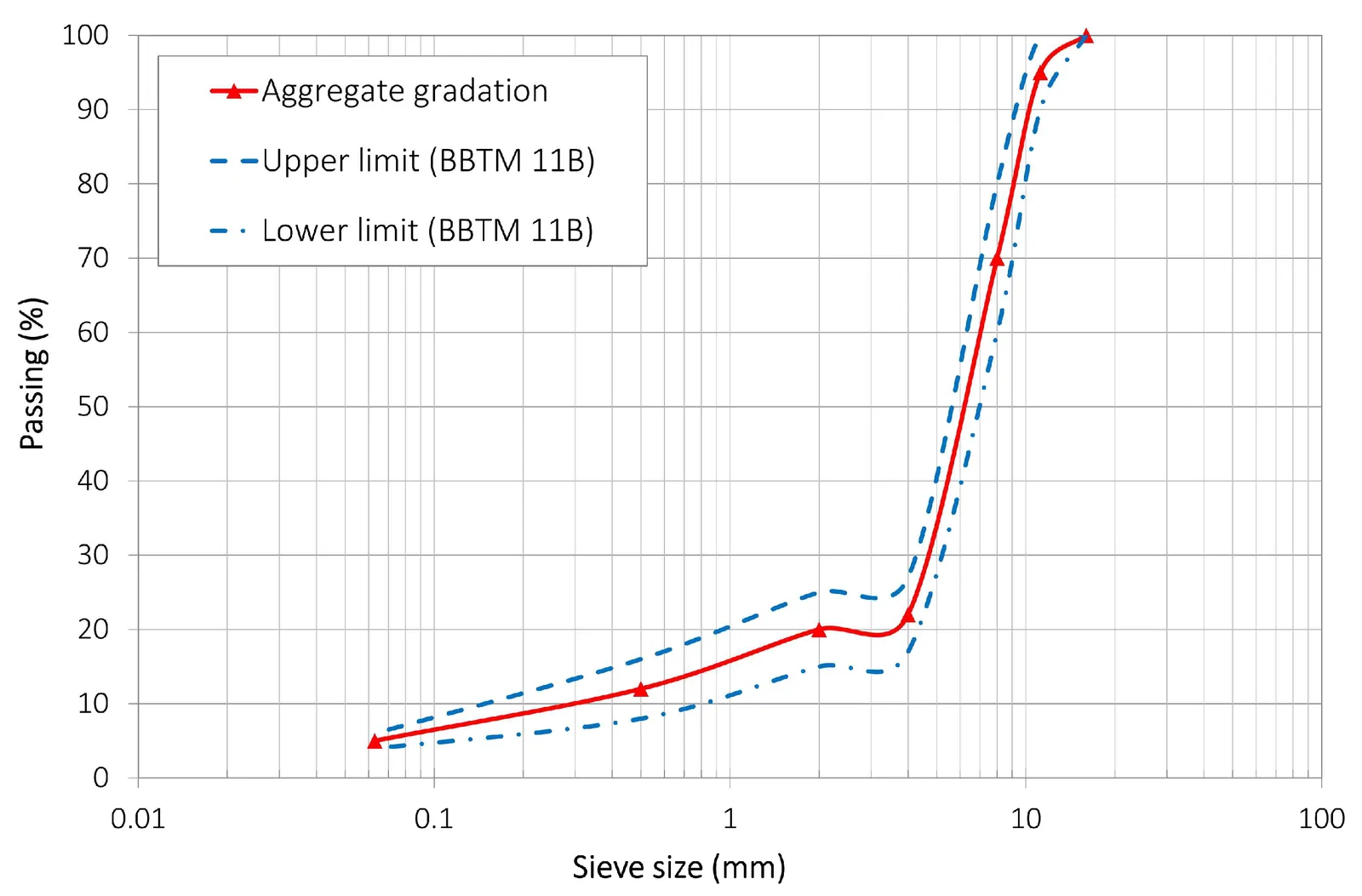
Aggregate gradation curve of the BBTM 11B asphalt mixture.

**Figure 3:**
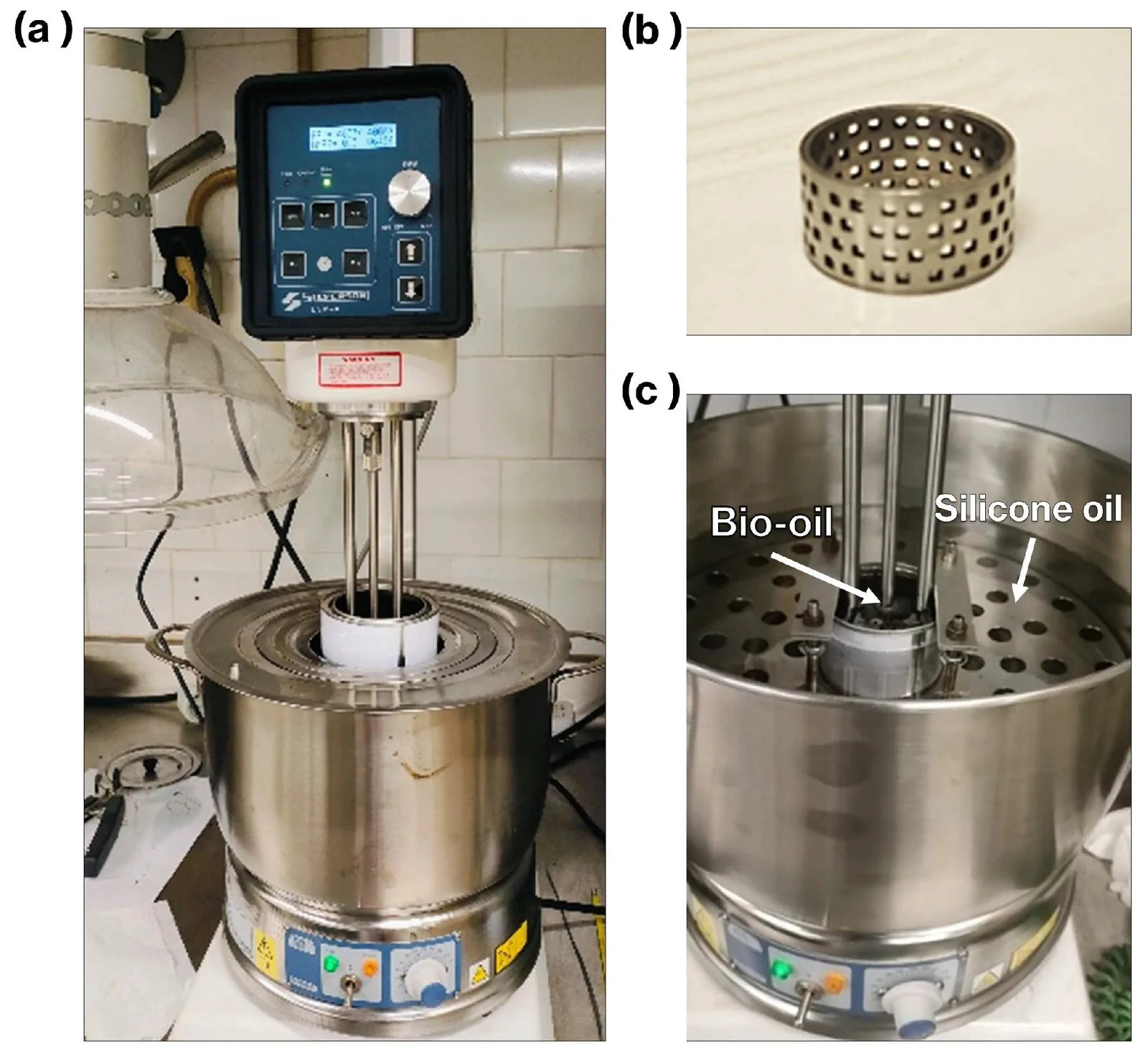
High-shear mixing unit with an adjustable thermal oil heating vessel: (**a**) high-shear laboratory mixer, (**b**) disintegrator head, and (**c**) heating bath setup.

**Figure 4:**
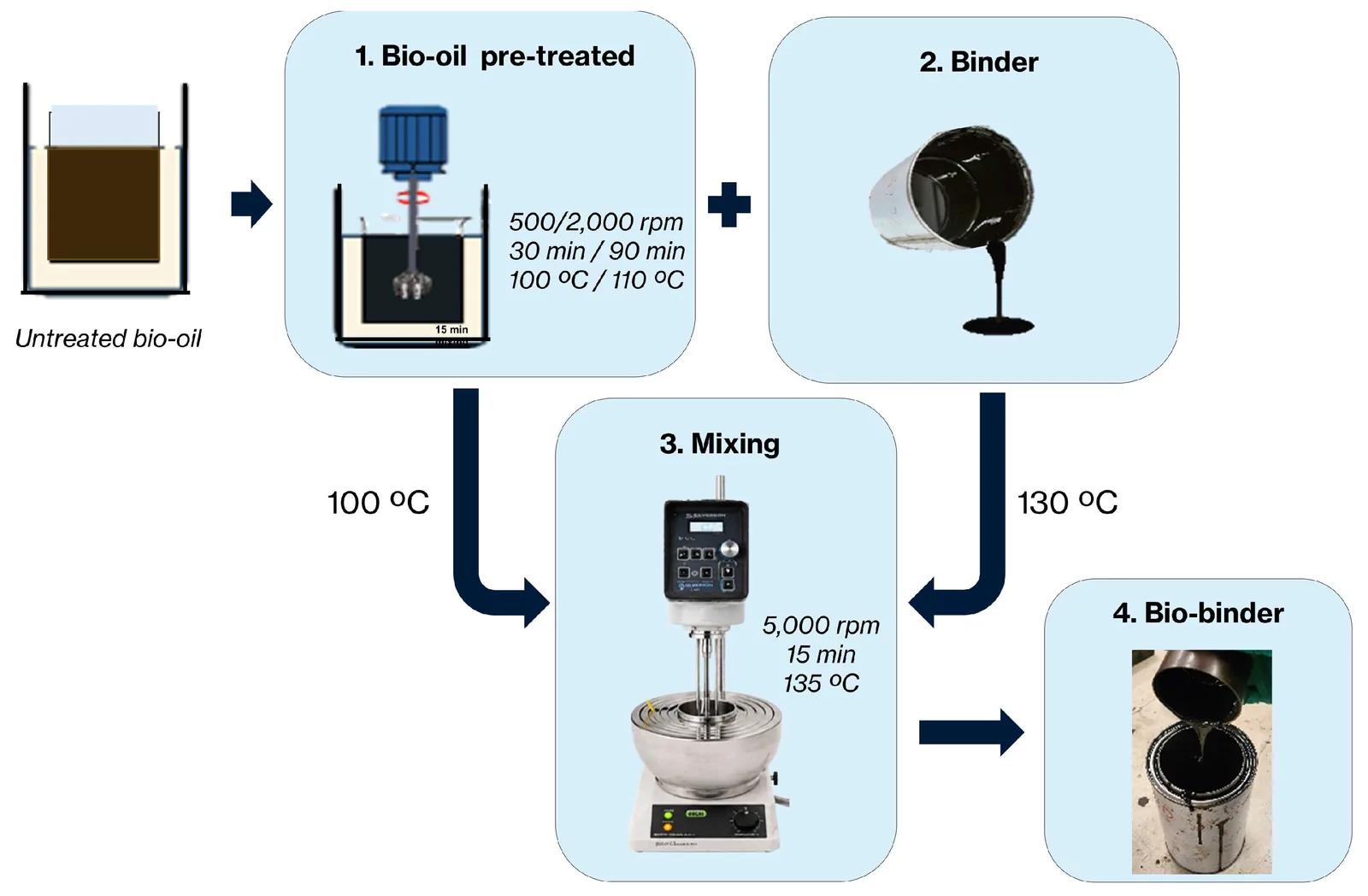
Mixing process for producing a bio-binder. The schematic illustration of the bio-oil pre-treatment process was generated using AI.

**Figure 5:**
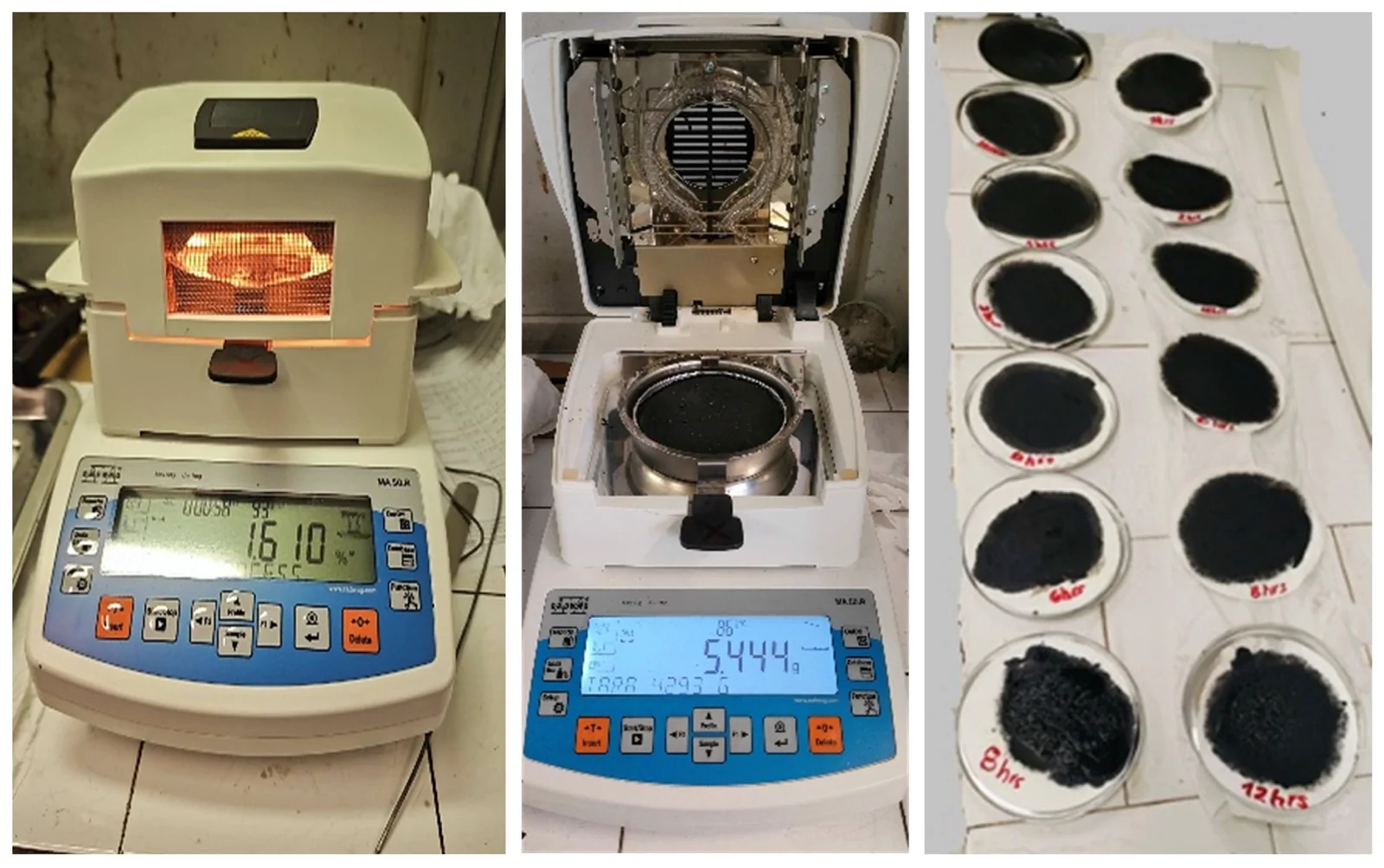
Thermobalance (left); tested bio-oil samples subjected to different pre-treatment durations (right).

**Figure 6:**
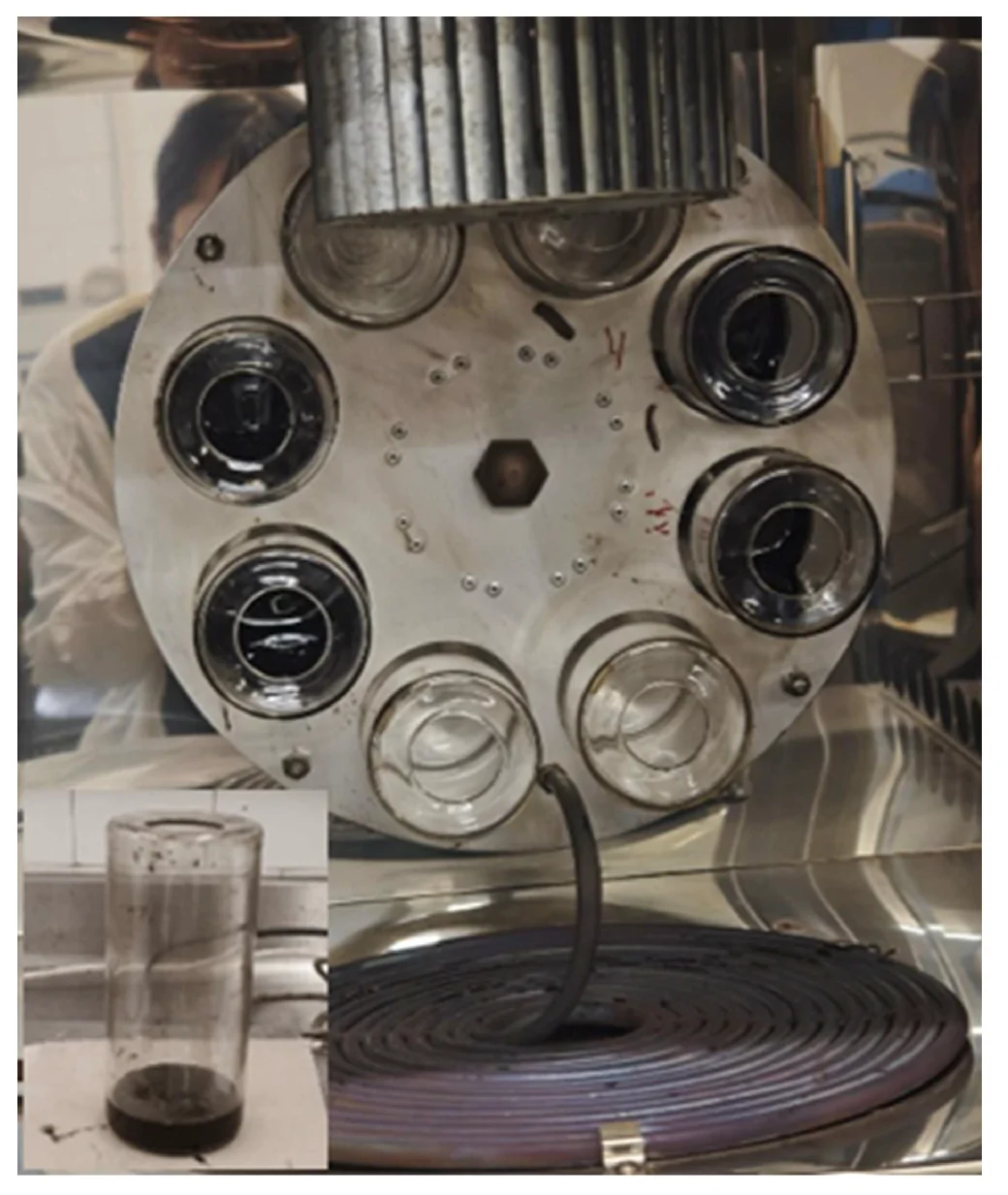
Binder samples during the short-term ageing test in the RTFOT oven.

**Figure 7:**
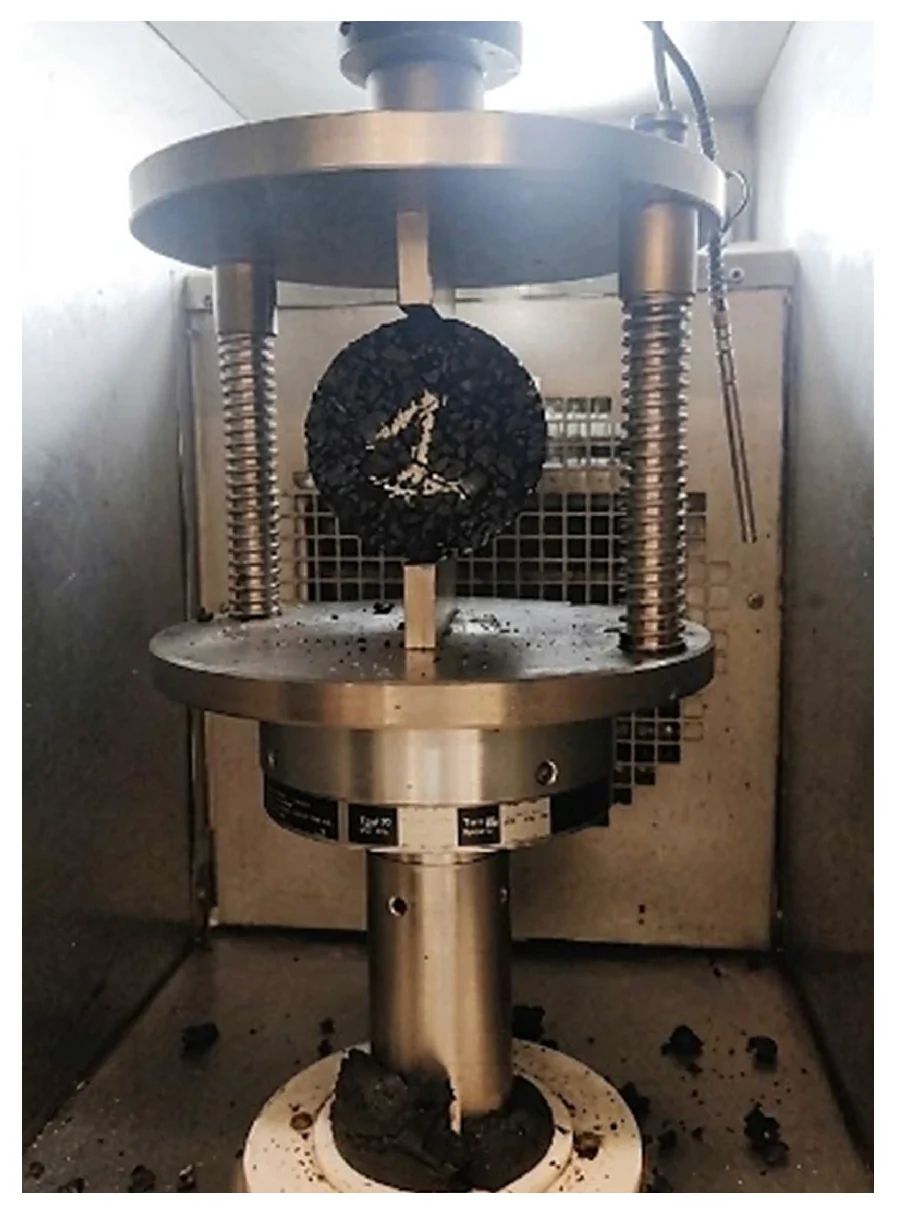
Specimens prepared for indirect tensile testing.

**Figure 8:**
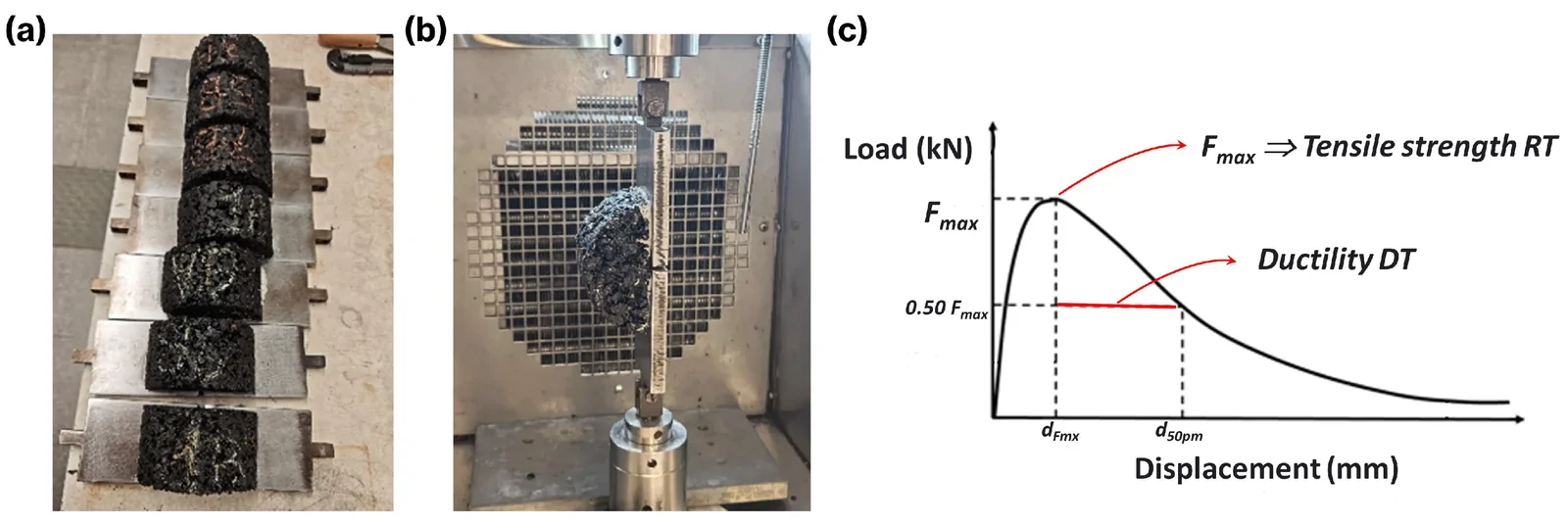
Fénix test procedure: (**a**) semi-circular test specimens prepared for the test, (**b**) specimen mounted on the Fénix device, and (**c**) a typical stress–strain curve obtained during the test [80].

**Figure 9:**
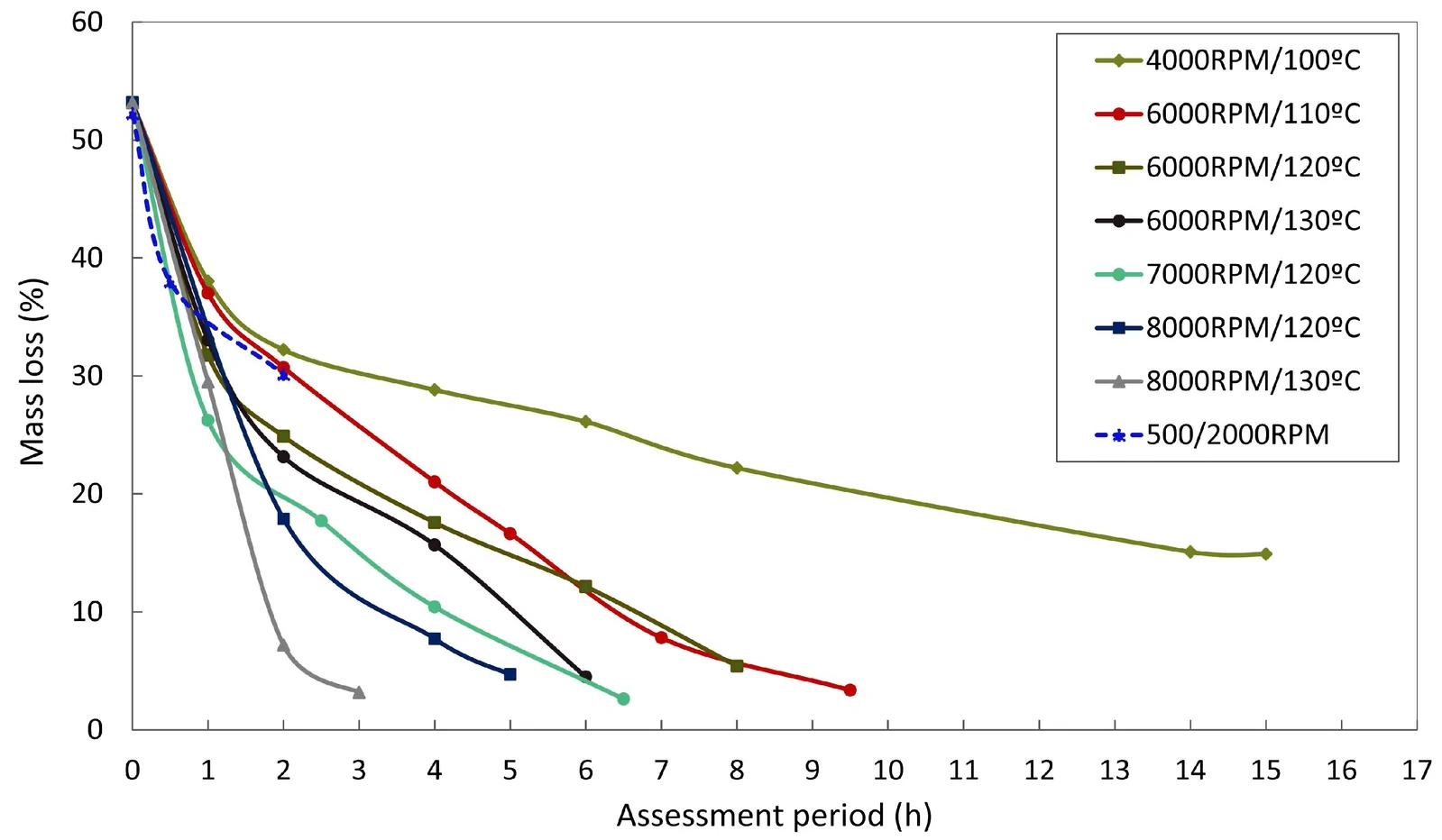
Changes in the residual mass loss of pre-treated bio-oil.

**Figure 10:**
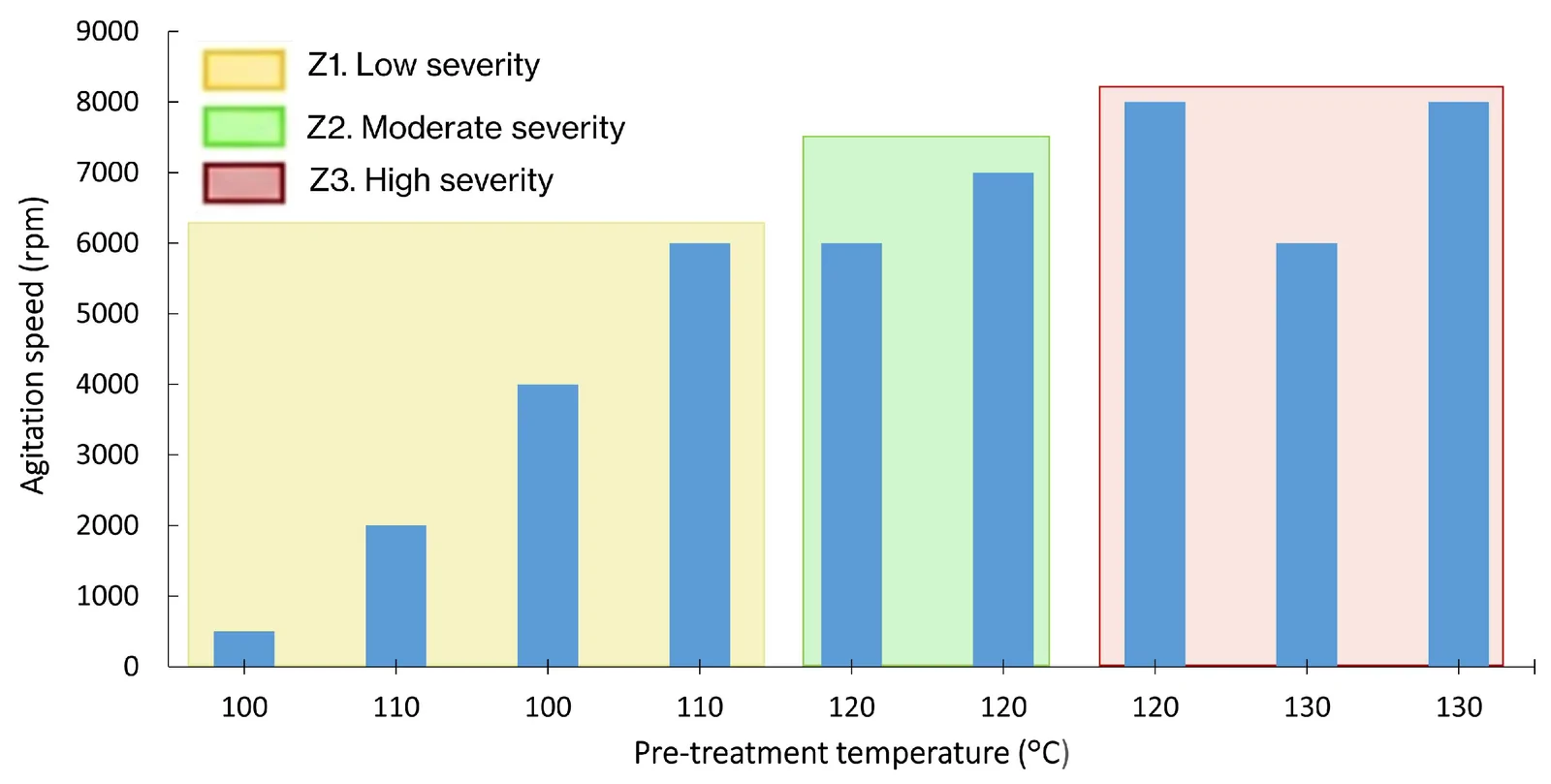
Map of operating windows as a function of pre-treatment temperature and stirring speed.

**Figure 11:**
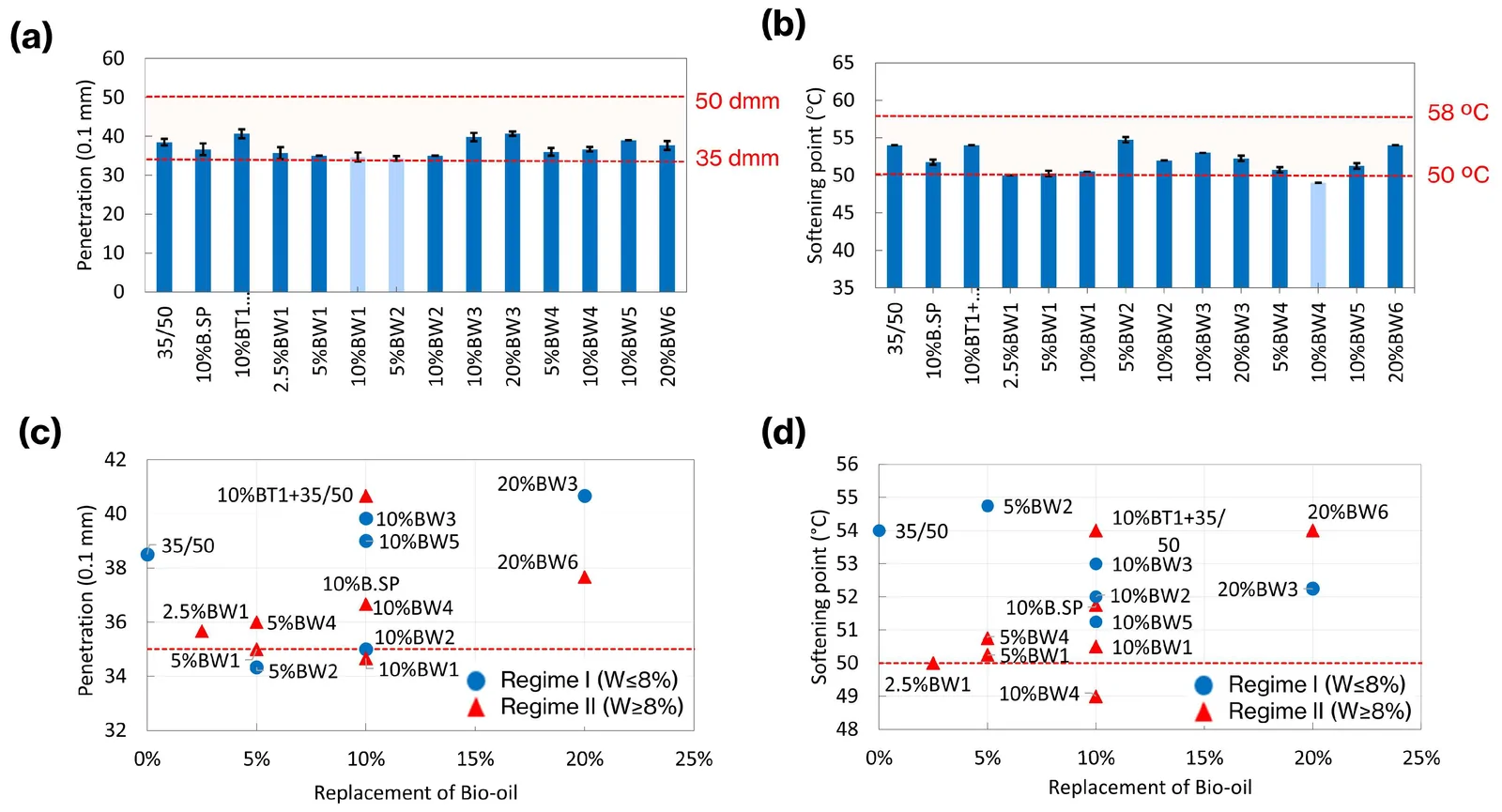
(**a**) Penetration and (**b**) softening point under initial conditions, (**c**) penetration versus bio-oil replacement (%), and (**d**) residual mass loss (ML) and softening point versus bio-oil replacement (%) and residual mass loss (ML).

**Figure 12:**
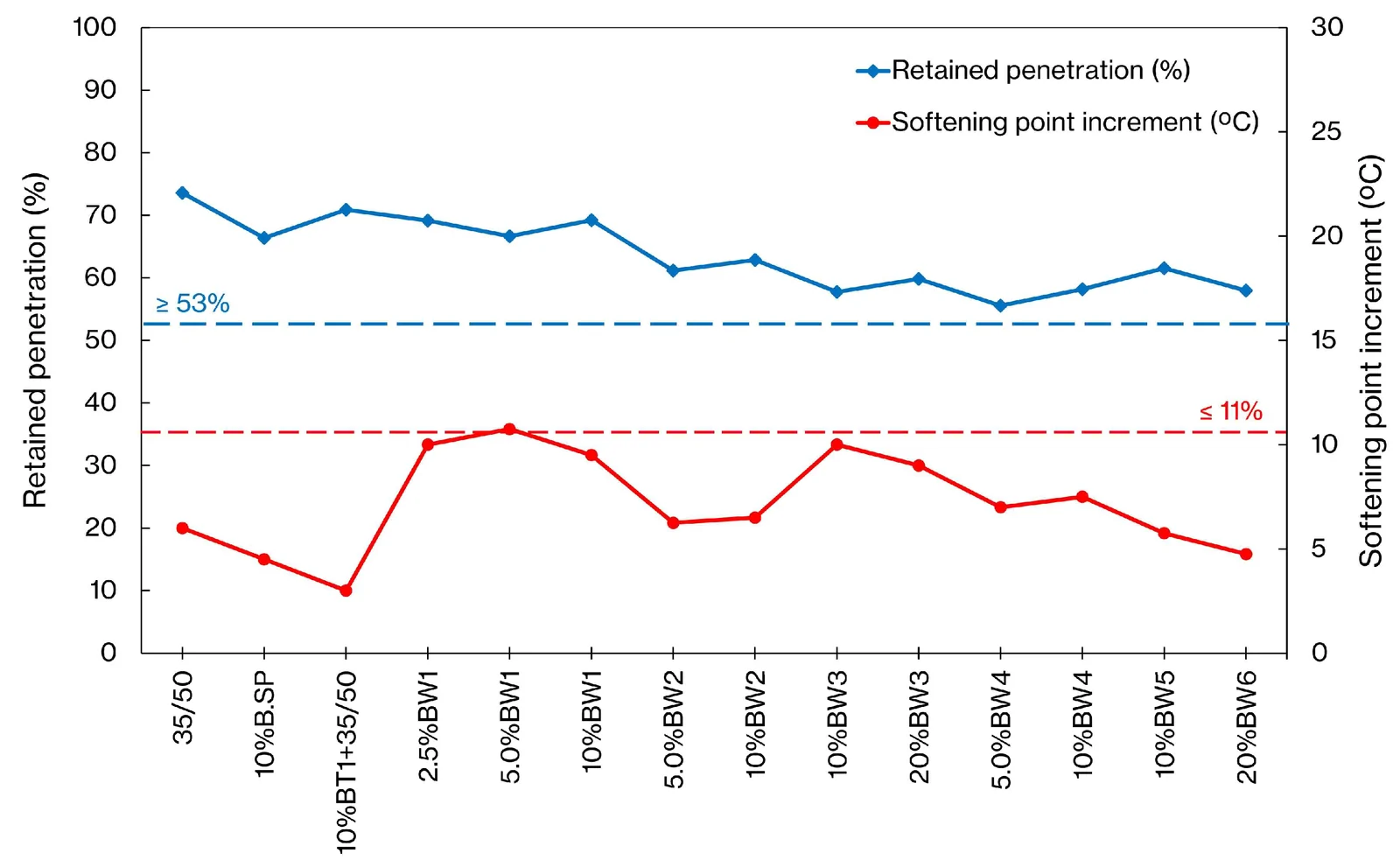
Susceptibility of bio-binders to ageing: retained penetration and increase in the softening point (RTFOT, EN 12607-1 [68]).

**Figure 13:**
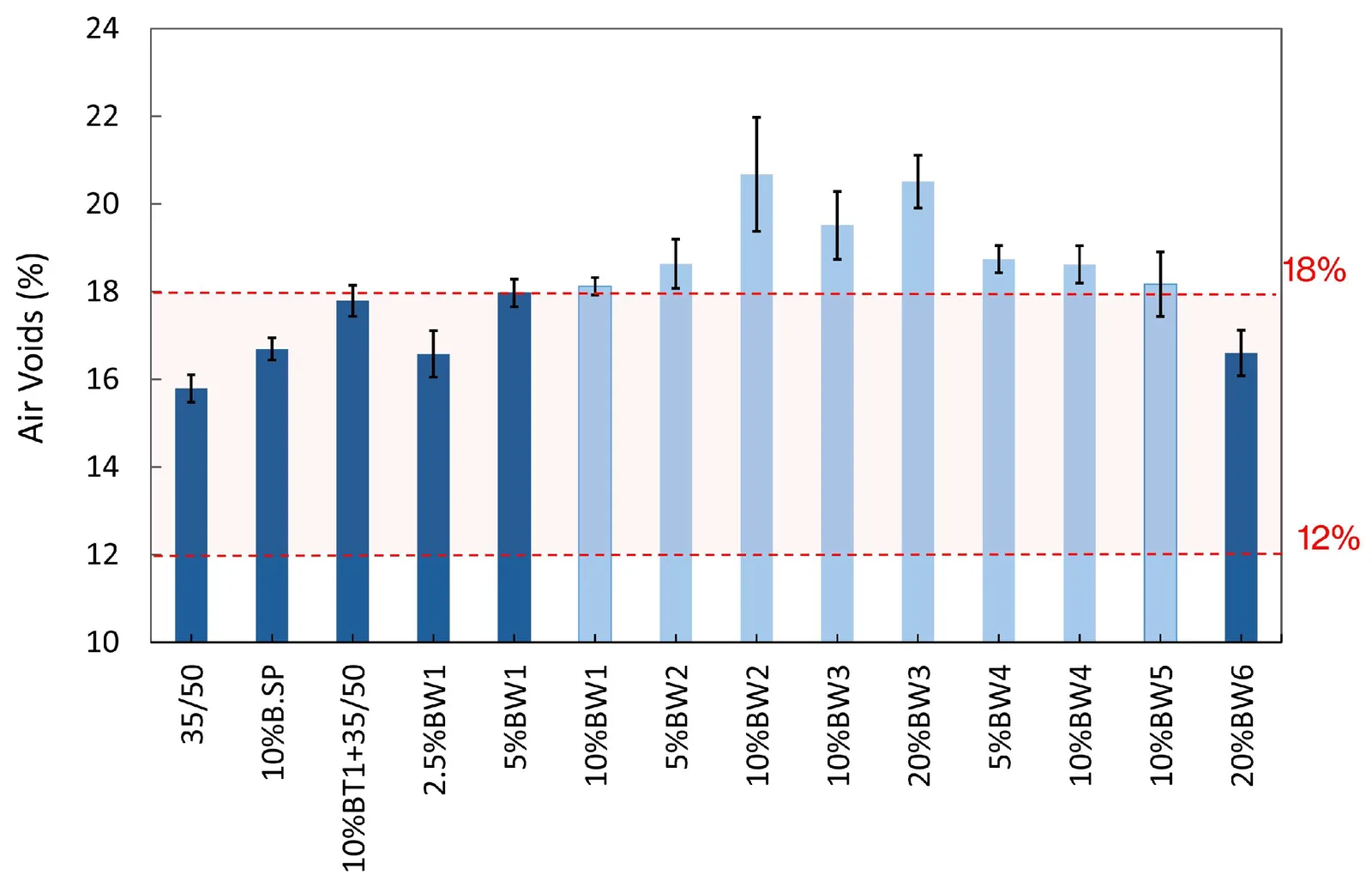
Air void content of BBTM 11B mixtures and the adopted PG-3 acceptance limits; measured according to EN 12697-8 [75].

**Figure 14:**
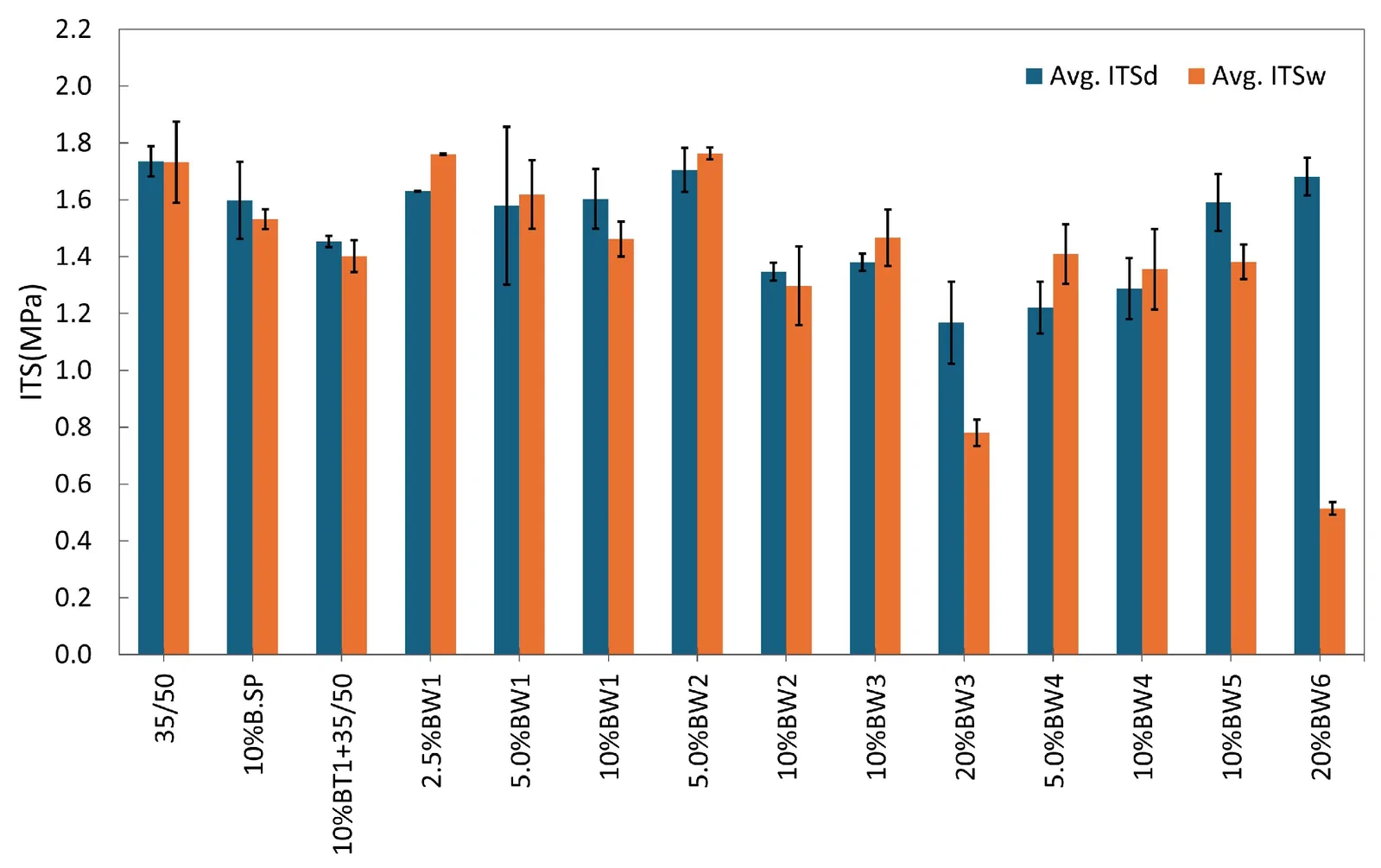
Mean values of indirect tensile strength (*ITS*).

**Figure 15:**
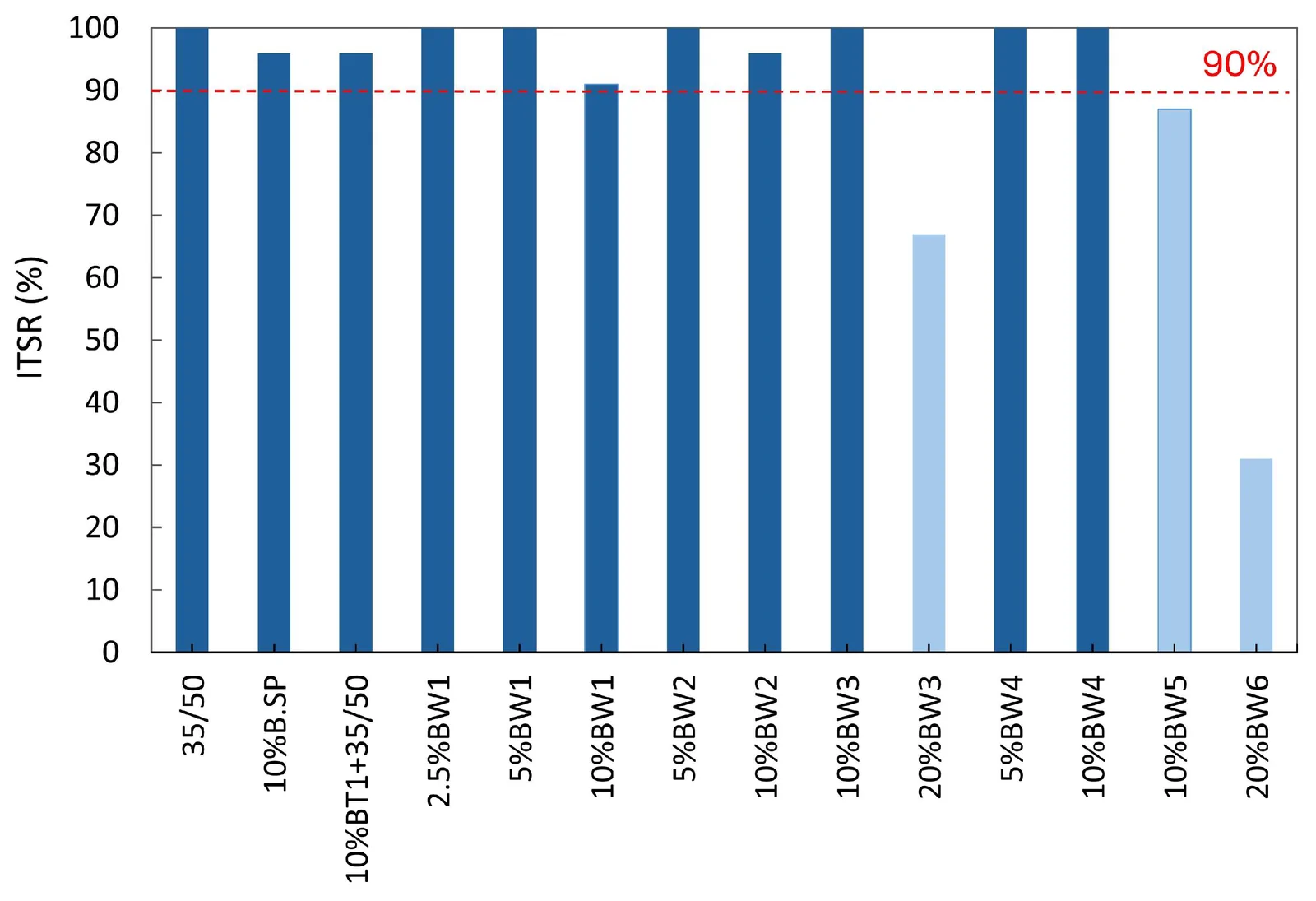
Indirect tensile strength ratio results.

**Figure 16:**
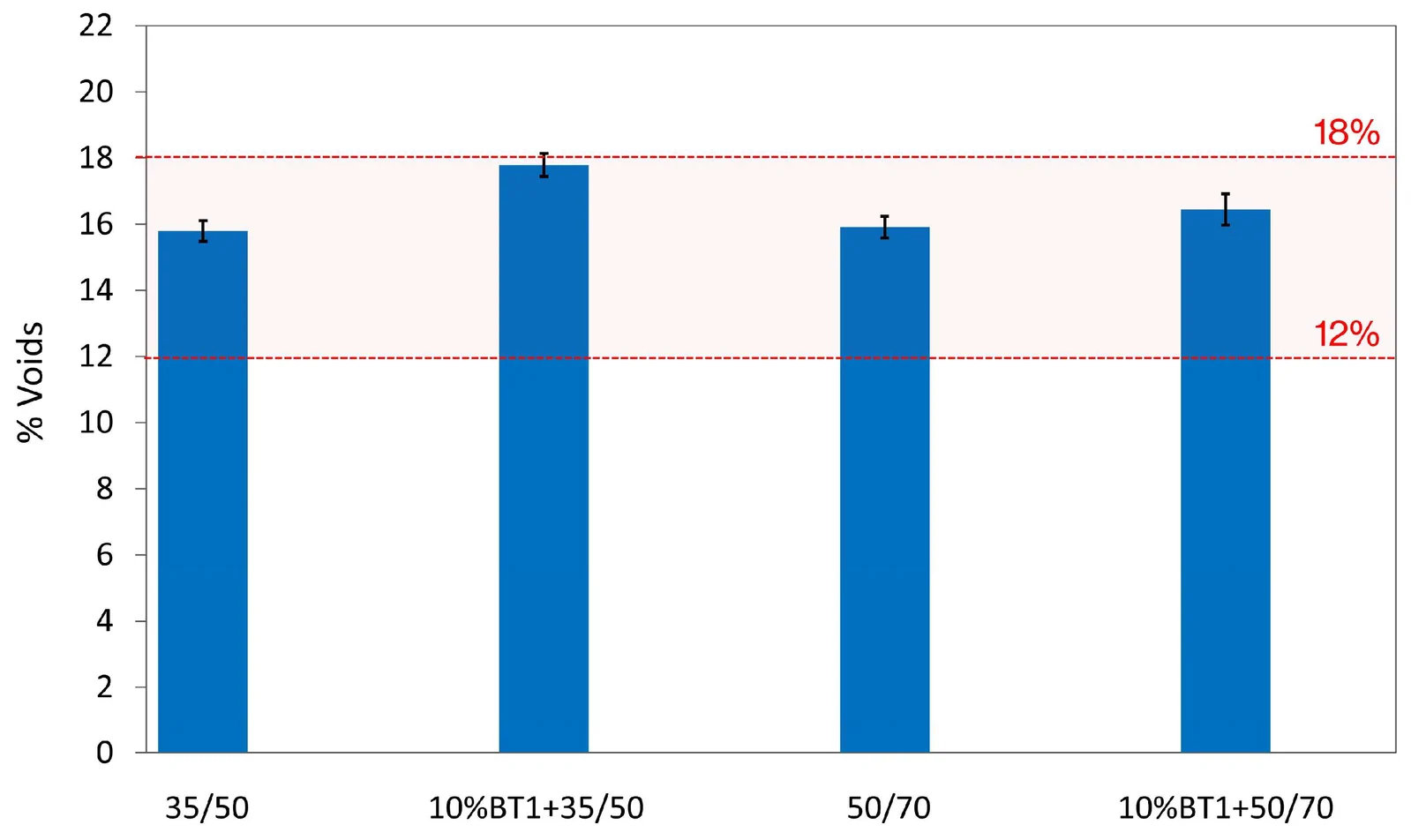
Air void content of the mixtures studied.

**Figure 17:**
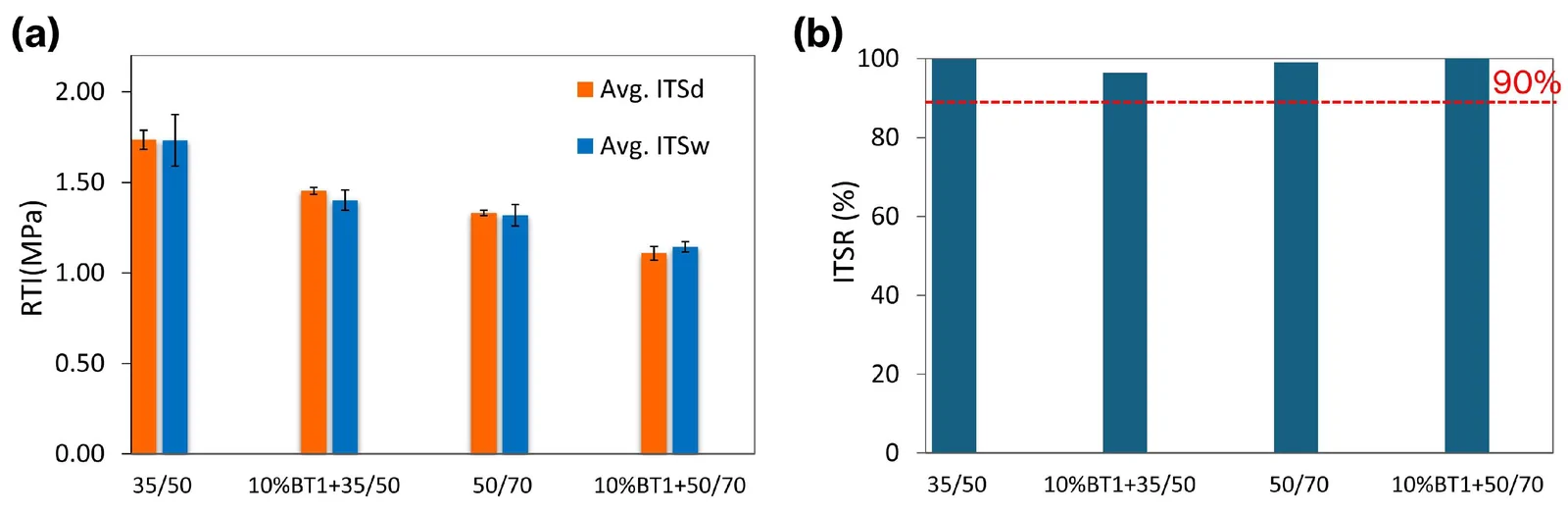
(**a**) Dry and wet indirect tensile strength and (**b**) indirect tensile strength ratio.

**Figure 18:**
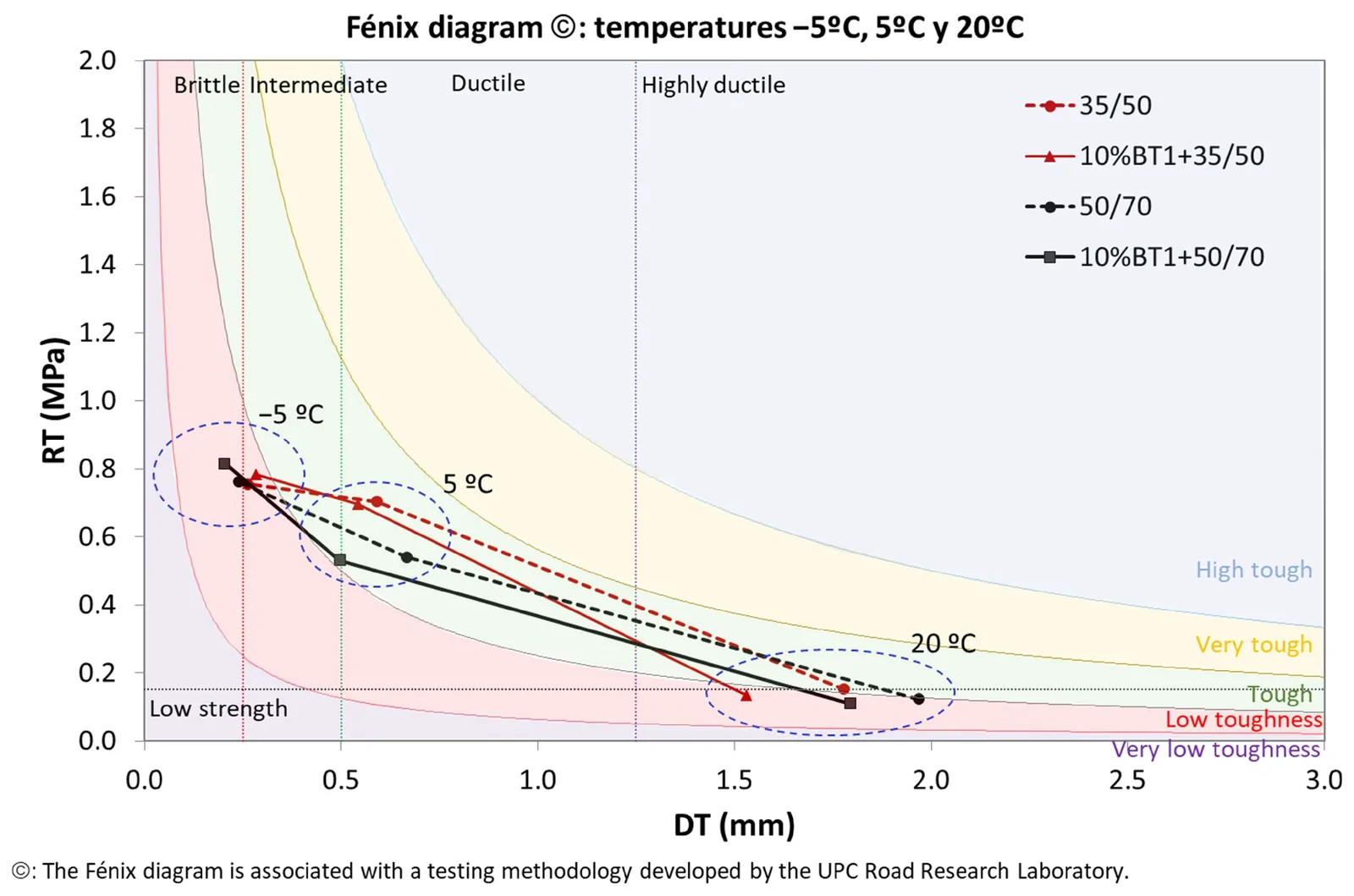
Fénix diagram.

**Table 2:** Matrix of experimental conditions and treatments durations for the series evaluated.

Series Code	Temperature (°C)	Stirring Speed (rpm)	Duration (h)
BT1 ^(1)^	100 + 110	500 + 2000	0.5 + 1.5
B2-T100-R4	100	4000	1.0; 2.0; 4.0; 6.0; 8.0; 14.0; 15.0
B3-T110-R6	110	6000	1.0; 2.0; 4.0; 5.0; 7.0; 9.5
B4-T120-R6	120	6000	1.0; 2.0; 4.0; 6.0; 8.0
B5-T130-R6	130	6000	1.0; 2.0; 4.0; 6.0
B6-T120-R7	120	7000	1.0; 2.5; 4.0; 6.5
B7-T120-R8	120	8000	1.0; 2.0; 4.0; 5.0
B8-T130-R8	130	8000	1.0; 2.0; 3.0

^(1)^ BT1: sequential treatment at 100 °C and 500 rpm for 0.5 h, followed by 110 °C and 2000 rpm for 1.5 h (total: 2 h).

**Table 3:** Experimental matrix of the prepared bio-binders.

Series Code	Bio-Oil Replacement	Sample Designation	Processing Conditions		
			Stirring Speed	Temperature	Time
	%		rpm	°C	h
Phase I: Characterisation of base materials and reference binders					
35/50 (reference)	N.A.	35/50	N.A.	N.A.	N.A.
50/70 (reference)	N.A.	50/70	N.A.	N.A.	N.A.
Unprocessed ^(1)^	10.0	10%B.SP	N.A.	N.A.	N.A.
Phase II: Exploratory study of dehydration under low to high shear (35/50 Matrix)					
B3-T110-R6	2.5	2.5%BW1	6000	110	5.0
B3-T110-R6	5.0	5.0%BW1	6000	110	5.0
B3-T110-R6	10.0	10%BW1	6000	110	5.0
B3-T110-R6	5.0	5.0%BW2	6000	110	7.0
B3-T110-R6	10.0	10%BW2	6000	110	7.0
B3-T110-R6	10.0	10%BW3	6000	110	9.5
B3-T110-R6	20.0	20%BW3	6000	110	9.5
B6-T120-R7	5.0	5.0%BW4	7000	120	2.5
B6-T120-R7	10.0	10%BW4	7000	120	2.5
B4-T120-R6	10.0	10%BW5	6000	120	8.0
B2-T100-R4	20.0	20%BW6	4000	100	15.0
B5-T130-R6 ^(2)^	---	---	6000	130	6.0
B7-T120-R8 ^(2)^	---	---	8000	120	5.0
B8-T130-R8 ^(2)^	---	---	8000	130	3.0
Phase III: Evaluation of the selected bio-binder with 35/50 and 50/70 bitumens					
BT1	10.0	10%BT1 + 35/50	500/2000 ^(3)^	100/110	2.0
BT1	10.0	10%BT1 + 50/70	500/2000 ^(3)^	100/110	2.0

^(1)^ Pure/crude bio-oil; ^(2)^ Discarded; ^(3)^ Sequential two-stage treatment (500 rpm for 0.5 h, followed by 2000 rpm for 1.5 h), total 2 h; N.A.: Not applicable. Note: B2–B8 and BT1 identify the initial bio-oil pre-treatment procedures (Table 2), whereas BW1–BW6 identify the selected pre-treatment conditions used for bio-binder preparation. The percentage preceding each code indicates the bio-oil replacement. B.SP refers to untreated bio-oil. Treatments B5, B7, and B8 were excluded. BT1 corresponds to a two-stage pre-treatment.

**Table 4:** Characteristics of the binders used in the BBTM 11B mixture.

Properties	Unit	Standard	35/50	Specification	50/70	Specification
Penetration at 25 °C	(0.1 mm)	EN 1426 [66]	38.5 ± 0.9	35–50	50.7 ± 0.6	50–70
Softening point	(°C)	EN 1427 [67]	54.0 ± 0.0	50–58	51.3 ± 0.4	46–54

**Table 5:** Reduction in measured mass loss recorded at the start and end of the test period for each series.

Series Code	Temperature (°C)	Stirring Speed (rpm)	Duration (h)	Residual Mass Loss (%)	Reduction in Mass Loss (%)	Comments
BT1	100 + 110	500 + 2000	0.5	38.0	29	
			2.0	30.1	43	
B2-T100-R4	100	4000	1.0	38.0	28	
			15	14.9	72	
B3-T110-R6	110	6000	1.0	37.0	30	
			9.5	3.4	94	
B4-T120-R6	120	6000	1.0	31.8	40	
			8.0	5.4	90	
B5-T130-R6	130	6000	1.0	33.1	38	Superficial degradation
			6.0	4.5	92	
B6-T120-R7	120	7000	1.0	26.2	51	
			6.5	2.6	95	
B7-T120-R8	120	8000	2.0	17.9	66	Severe degradation
			5.0	4.7	91	
B8-T130-R8	130	8000	1.0	29.5	45	Severe degradation
			3.0	3.2	94	

**Table 6:** Mass loss results obtained using the RTFOT method (EN 12607-1 [68]).

Sample Designation	Bio-Oil Replacement (%)	W_bio-binder_ Before Ageing (%)	RTFOT Mass Loss (%)	Standard Criterion (EN 12607-1 [68]) for 35/50 Bitumen
35/50	0.0	0.0	0.05 ± 0.00	Compliant
10%B.SP	10.0	2.2	0.18 ± 0.01	Compliant
10%BT1 + 35/50	10.0	2.1	0.26 ± 0.02	Compliant
2.5%BW1	2.5	0.0	0.44 ± 0.03	Compliant
5.0%BW1	5.0	0.4	0.44 ± 0.03	Compliant
10%BW1	10.0	0.6	0.47 ± 0.03	Compliant
5.0%BW2	5.0	0.2	0.40 ± 0.03	Compliant
10%BW2	10.0	0.0	0.31 ± 0.02	Compliant
10%BW3	10.0	0.0	0.02 ± 0.00	Compliant
20%BW3	20.0	0.0	1.04 ± 0.07	Non-compliant
5.0%BW4	5.0	0.0	0.17 ± 0.01	Compliant
10%BW4	10.0	0.4	1.34 ± 0.09	Non-compliant
10%BW5	10.0	0.0	1.68 ± 1.02	Non-compliant
20%BW6	20.0	0.0	0.99 ± 0.07	Non-compliant

**Table 7:** Compliance matrix of bio-modified binders and asphalt mixtures against specification limits.

Nomenclature	Penetration (35–50 dmm)	Softening Point (50–58 °C)	Retained Penetration (≥53%)	Softening Point Increment (≤11 °C)	Mass Change (≤0.5%)	Air Voids (12–18%)	*ITSR* (≥90%)	Failed Criteria	Global Compliance
35/50	38.5	54.0	73.6	6.0	0.05	15.8	100	0	PASS
10%B.SP	36.7	51.8	66.4	4.5	0.18	16.7	96	0	PASS
10%BT1 + 35/50	40.7	54.0	70.9	3.0	0.26	17.8	96	0	PASS
2.5%BW1	35.7	50.0	69.2	10.0	0.44	16.6	108	0	PASS
5.0%BW1	35.0	50.3	66.7	10.8	0.44	18.0	102	0	PASS
10%BW1	34.7	50.5	69.2	9.5	0.47	18.1	91	2	FAIL
5.0%BW2	34.3	54.8	61.2	6.3	0.40	18.6	103	2	FAIL
10%BW2	35.0	52.0	62.9	6.5	0.31	20.7	96	1	FAIL
10%BW3	39.8	53.0	57.7	10.0	0.02	19.5	106	1	FAIL
20%BW3	40.7	52.3	59.8	9.0	1.04	20.5	67	3	FAIL
5.0%BW4	36.0	50.8	55.6	7.0	0.17	18.7	115	1	FAIL
10%BW4	36.7	49.0	58.2	7.5	1.34	18.6	105	3	FAIL
10%BW5	39.0	51.3	61.5	5.8	1.68	18.2	87	3	FAIL
20%BW6	37.7	54.0	58.0	4.8	0.99	16.6	31	2	FAIL

**Table 8:** Physical properties of the bio-binders studied before and after RTFOT ageing.

Properties	Standard	Limits	35/50	10%BT1 + 35/50	Limits	50/70	10%BT1 + 50/70
Before ageing							
Penetration at 25 °C (0.1 mm)	EN 1426 [66]	35–50	38.5 ± 0.9	40.7 ± 1.2	50–70	50.7 ± 0.6	58.0 ± 1.7
Softening point (°C)	EN 1427 [67]	50–58	54.0 ± 0.0	54.0 ± 0.0	46–54	51.3 ± 0.4	54.0 ± 0.0
Bio-binder mass loss (%)	---	-	0.0	2.1	-	0.0	2.2
After RTFOT ageing (EN 12607-1 [68])							
Mass change (%)	EN 12607-1 [68]	≤0.5	0.05 ± 0.00	0.26 ± 0.02	≤0.5	0.29 ± 0.02	0.19 ± 0.01
Retained penetration (%)	EN 1426 [66]	≥53	73.6 ± 2.2	70.9 ± 2.1	≥53	69.4 ± 2.6	66.7 ± 2.8
Increase in softening point (°C)	EN 1427 [67]	≤11.0	6.0 ± 0.0	3.0 ± 0.0	≤10.0	6.8 ± 0.0	4.3 ± 0.0

**Table 9:** Average parameters resulting from the Fénix test.

Temperature (°C)	35/50		10%BT1 + 35/50		50/70		10%BT1 + 50/70	
	*RT* (MPa)	*DT* (mm)	*RT* (MPa)	*DT* (mm)	*RT* (MPa)	*DT* (mm)	*RT* (MPa)	*DT* (mm)
20	0.15 ± 0.03	1.78 ± 0.05	0.13 ± 0.00	1.53 ± 0.03	0.12 ± 0.01	1.97 ± 0.08	0.11 ± 0.02	1.79 ± 0.11
5	0.70 ± 0.01	0.59 ± 0.09	0.70 ± 0.04	0.54 ± 0.10	0.54 ± 0.03	0.67 ± 0.06	0.53 ± 0.02	0.50 ± 0.05
−5	0.75 ± 0.03	0.26 ± 0.08	0.78 ± 0.00	0.28 ± 0.08	0.76 ± 0.08	0.24 ± 0.18	0.82 ± 0.00	0.20 ± 0.05

## Data Availability

The original contributions presented in this study are included in the article. Further inquiries can be directed to the corresponding author.
